# Single-arm trial of neoadjuvant ipilimumab plus nivolumab with chemoradiotherapy in patients with resectable and borderline resectable lung cancer: the INCREASE study

**DOI:** 10.1136/jitc-2024-009799

**Published:** 2024-09-30

**Authors:** Idris Bahce, Chris Dickhoff, Famke L Schneiders, Joris Veltman, David J Heineman, Sayed M S Hashemi, Anne Vrijmoet, Ilias Houda, Ezgi B Ulas, Joyce Bakker, Peter van de Ven, Natalja Bouwhuis, Lilian J Meijboom, Daniela E Oprea-Lager, Febe van Maldegem, Marieke F Fransen, Tanja D de Gruijl, Teodora Radonic, Suresh Senan

**Affiliations:** 1 Department of Pulmonary Medicine, Amsterdam UMC Location VUmc, Amsterdam, Noord-Holland, Netherlands; 2 Imaging and Biomarkers, Cancer Centre Amsterdam, Amsterdam, Noord-Holland, Netherlands; 3 Department of Cardiothoracic Surgery, Amsterdam UMC location Vrije Universiteit Amsterdam, Amsterdam, Netherlands; 4 Department of Radiation Oncology, Amsterdam UMC location Vrije Universiteit Amsterdam, Amsterdam, Netherlands; 5 Department of Pulmonary Medicine, Amsterdam UMC location Vrije Universiteit Amsterdam, Amsterdam, Netherlands; 6 Department of Pulmonary Medicine, location Vrije Universiteit Amsterdam, Amsterdam, Netherlands; 7 Department of Pulmonary Medicine, Amsterdam UMC location Vrije Universiteit Amsterdam, Amsterdam, Noord-Holland, Netherlands; 8 Department of Pulmonary Medicine, Amsterdam UMC Locatie VUmc, Amsterdam, Netherlands; 9 Department of Medical Oncology, Amsterdam UMC location Vrije Universiteit Amsterdam, Amsterdam, Netherlands; 10 Department of Epidemiology & Biostatistics, Amsterdam UMC location Vrije Universiteit Amsterdam, Amsterdam, Netherlands; 11 Department of Clinical Pharmacology and Pharmacy, Amsterdam UMC location Vrije Universiteit Amsterdam, Amsterdam, Netherlands; 12 Department of Radiology and Nuclear Medicine, Amsterdam UMC location Vrije Universiteit Amsterdam, Amsterdam, Netherlands; 13 Radiology and Nuclear Medicine, Amsterdam UMC Locatie VUmc, Amsterdam, Netherlands; 14 Molecular Cellular Biology and Immunology, Amsterdam UMC Location VUmc, Amsterdam, Netherlands; 15 Cancer Center Amsterdam, Amsterdam, Netherlands; 16 Department of Pathology, Amsterdam UMC location Vrije Universiteit Amsterdam, Amsterdam, Noord-Holland, Netherlands; 17 Department of Radiation Oncology, Amsterdam UMC Locatie VUmc, Amsterdam, Noord-Holland, Netherlands

**Keywords:** Nivolumab, Ipilimumab, Chemotherapy, Radiotherapy

## Abstract

**Background:**

In non-small cell lung cancer (NSCLC), chemoradiotherapy (CRT) yields pathological complete response (pCR) rates of approximately 30%. We investigated using ipilimumab plus nivolumab (IPI-NIVO) with neoadjuvant CRT in resectable, and borderline resectable NSCLC.

**Methods:**

This single-arm, phase-II trial enrolled operable T3-4N0–2 patients with NSCLC without oncogenic drivers. Primary study endpoints were safety, major pathological response (MPR) and pCR. Treatment encompassed platinum-doublet concurrent CRT, IPI 1 mg/kg intravenous and NIVO 360 mg intravenous on day-1, followed by chemotherapy plus NIVO 360 mg 3 weeks later. Thoracic radiotherapy was 50 or 60 Gy, in once-daily doses of 2 Gy. Resections were 6 weeks post-radiotherapy.

**Results:**

In a total of 30 patients in the intention-to-treat (ITT) population, grades 3–4 treatment-related adverse events (TRAEs) occurred in 70%, one TRAE grade 5 late-onset pneumonitis on day 96 post-surgery (1/30, 3.3%) occurred, and one non-TRAE COVID-19 death (1/30, 3.3%). pCR and MPR were achieved in 50% (15/30) and 63% (19/30) of the ITT; and in 58% (15/26) and 73% (19/26) of the 26 patients who underwent surgery, respectively. Postoperative melanoma was seen in one non-pCR patient. The R0 rate was 100% (26/26), and no patient failed surgery due to TRAEs. In peripheral blood, proliferative CD8^+^ T cells were increased, while proliferative regulatory T cells (Tregs) were not. On-treatment, pCR-positives had higher CD8^+^CD39^+^ T cells and lower HLA-DR^+^ Tregs.

**Conclusions:**

Neoadjuvant IPI-NIVO-CRT in T3-4N0–2 NSCLC showed acceptable safety with pCR and MPR in 58% and 73% of operated patients, respectively. No patient failed surgery due to TRAEs.

**Trial registration number:**

NCT04245514.

**WHAT IS ALREADY KNOWN ON THIS TOPIC**

- Patients with locally advanced borderline resectable non-small cell lung cancer (NSCLC), treated with chemoradiotherapy followed by surgery, experience significant local and distant disease relapse.

**WHAT THIS STUDY ADDS**

- INCREASE added ipilimumab and nivolumab to standard chemoradiotherapy and surgery in borderline resectable NSCLC, achieving ca 60% pathological complete response (pCR) compared with the historical 30%, with no surgery failures due to treatment-related adverse events.

**HOW THIS STUDY MIGHT AFFECT RESEARCH, PRACTICE OR POLICY**

- These findings suggest that adding dual immunotherapy could enhance the efficacy of current standard care for locally advanced borderline resectable NSCLC. Further research is needed to determine the optimal combination of treatments for maximum efficacy with minimal toxicity.

## Introduction

 Non-small cell lung cancer (NSCLC) accounts for the highest incidence of cancer-related death worldwide.^1^ A subgroup of patients with NSCLC with large tumors, or tumors invading the chest wall or mediastinum (T3-T4), either with or without nodal involvement (N0-N2), can undergo surgery either at initial presentation, or following induction therapy. In selected patients, guidelines recommend resection after induction chemoradiotherapy (CRT) in order to maximize local and distant control rates.^2 3^ The use of multimodal strategies involving platinum-doublet chemotherapy combined with radiotherapy has improved long-term clinical outcomes, with acceptable toxicity.^24^,^7^ However, overall survival (OS) is impaired by distant disease relapses which underlines the need for improved systemic tumor control.^8^,^10^

Neoadjuvant immunotherapy can improve local and distant control rates in patients presenting with resectable NSCLC. A phase-II trial compared neoadjuvant ipilimumab (anti-cytotoxic T-lymphocyte associated protein 4 (CTLA-4), IPI) plus nivolumab (anti-programmed cell death protein-1 (PD-1), NIVO) to neoadjuvant NIVO alone, in patients with stages I-IIIA NSCLC, and reported pathological complete response rates (pCR) of 29% with IPI-NIVO and 9% with NIVO alone in the intention-to-treat (ITT) population, and in the resected patient population: 38% with IPI-NIVO and 10% with NIVO alone.^11^ Furthermore, synergy may arise from combining immunotherapy with radiotherapy.^12^ These findings suggested that antitumor immune activity could be enhanced through synergy between radiotherapy and anti-CTLA-4 therapy.

We postulated that adding IPI-NIVO to CRT would further enhance pCR rates and antitumor immune responses. Therefore, the aim of this study was to investigate the addition of IPI-NIVO to standard induction CRT in patients with resectable and borderline resectable T3-4N0-2 stage IIB-III NSCLC tumors to determine safety and pathological response.

## Materials and methods

### Study population and design

#### Participants and screening

Between January 2020 and July 2022, this single-center, single-arm prospective phase-II study (INCREASE trial) enrolled fit patients with pathology-proven cT3-4N0-2M0 NSCLC according to the tumor, node, metastases eighth edition.^13^ Patients with actionable genomic alterations like activating EGFR or BRAF mutations or ALK or ROS1 gene rearrangements were excluded. A list of tested genomic alterations is provided in online supplemental table 1. Staging was performed according to European Society for Medical Oncology (ESMO) guidelines, including a tumor biopsy, pulmonary function tests, an ^18^F-fluorodeoxyglucose (FDG) positron emission tomography (PET)/CT scan, mediastinal staging using EBUS and brain imaging.^2^ No patient underwent a mediastinoscopy. At two time points, a decision on resectability was taken within the multidisciplinary team (MDT) by at least two experienced surgeons, namely: during screening and after finishing CRT. Criteria for resectability included cT3-4N0-2, excluding those T3-4 based on satellite lung lesions, those based on ingrowth in the heart, aorta, trachea, and esophagus, and excluding multistation N2. The criteria for operability were determined based on the preoperative risk assessment tests recommended by ESMO.^2^ The clinical trial protocol, including the full list of inclusion and exclusion criteria, has been published.^14^

### Procedures overview

A detailed description of the pre-surgical and post-surgical diagnostic and treatment procedures are given in the online supplemental data (chapter 1). In brief, patients received two cycles of chemotherapy concurrent with thoracic radiotherapy to a dose of 50 Gy, delivered in once-daily fractions of 2 Gy. For cases judged to be “borderline resectable” by surgeons, the total radiation dose delivered was 60 Gy.^6^ On day-1 of chemotherapy, one cycle of IPI (1 mg/kg intravenous) plus NIVO (360 mg flat dose intravenous) (IPI/NIVO) was administered. A second cycle of NIVO (360 mg flat dose intravenous) plus chemotherapy was administered 3 weeks later.

### Clinical outcomes

The co-primary study objectives were to assess the (1) safety and (2) pathological response using major pathological response (MPR) and pCR in the ITT population and in the evaluable patients population, that is, those who completed induction therapy, including at least one cycle of immunotherapy, followed by surgery.^15^ Secondary objectives were to assess disease-free survival (DFS), defined as the time from resection to recurrence of tumor or death, and OS, defined as the time from study inclusion to death. Exploratory objectives included assessing the immune competence of tumor-draining lymph nodes, and changes in peripheral blood immune subset distributions and phenotypes. The definitions used for primary, secondary and exploratory endpoints were as reported in our previously published trial protocol and are also described in the online supplemental data (chapter 2).^14^

### Tumor tissue, peripheral blood mononuclear cell analyses

**Figure 1:**
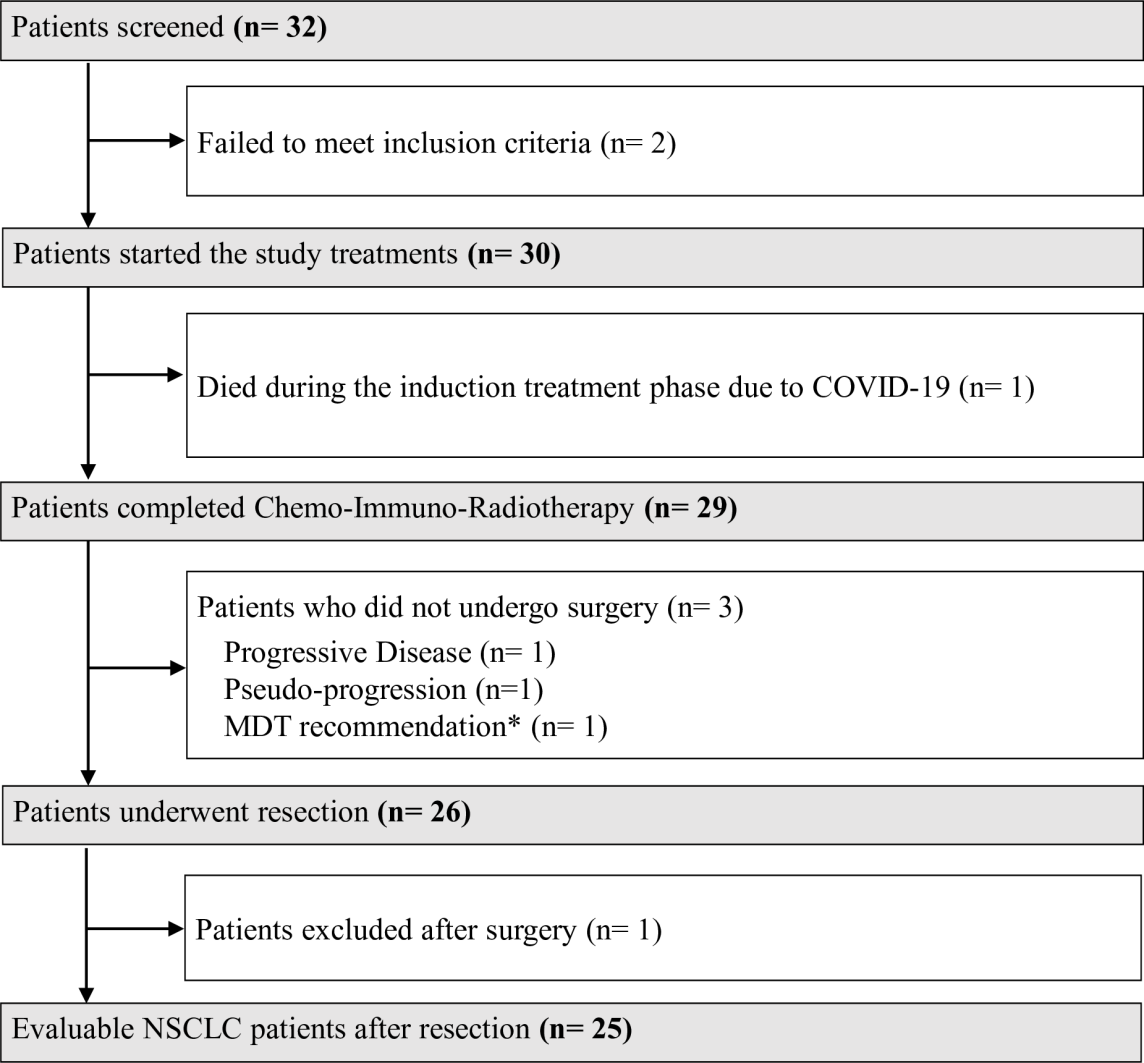
The patient disposition in the INCREASE trial. (*) This patient achieved a metabolic complete response after induction chemo-immuno-radiotherapy and the MDT recommended to omit surgery that would have involved a most likely futile resection of three vertebrae. MDT, multidisciplinary tumor board; NSCLC, non-small cell lung cancer.

A detailed description of the procedures regarding the pathological evaluation of tumor response, programmed death-ligand 1 (PD-L1), tumor genomic analysis, peripheral blood mononuclear cell (PBMC) analyses, and the depiction of the gating strategy and the specific markers used, including those for the regulatory T cells (Treg) population, is included in the (online supplemental data chapter 3, figure 1, tables 1,2).

### Statistical analysis

Per the study protocol, the co-primary aims were safety and assessing pathological response, and both pCR and MPR were considered as endpoints. However, as pCR has been more consistently reported in published papers for “historical” comparison, and as pCR is less prone to differing assessment criteria and interobserver variability, we decided to choose pCR to estimate our sample size and report as main efficacy endpoint.^6 8 9^

As compared with the previously reported rates of pCR following CRT averaging around 30%, this study aimed to test whether the pCR rate would double to 60%, using a Z-test for a single proportion with a two-sided significance level of 5%. A total of 26 evaluable patients were needed to reach 90% power to reject the null hypothesis if pCR after IPI-NIVO-CRT was 60%. To account for a drop-out rate of 10%, the planned accrual was 29 patients.

Descriptive statistics (proportions with 95% CIs) were used to summarize the endpoints, using frequencies and percentages for categorical variables and as median and range for continuous variables. Differences between categorical and continuous variables were assessed by t-test or χ^2^ test, when appropriate. For translational analyses with multiple measurements, two-way analysis of variance was used to compare pCR and non-pCR patients per time point; corrections for multiple comparisons were performed according to either Tukey or Šidàk, when appropriate. Detailed results with p values are provided in the online supplemental data (chapter 6).

## Results

### Patients disposition and characteristics

30 patients commenced induction therapy, of which 26 (87%) underwent post-induction surgery (see figure 1). Four patients did not undergo surgery, which in one case was due to death from respiratory failure due to a COVID-19 infection during induction therapy. Three patients failed to undergo surgery because of disease progression (N=1), new lung nodules that were later determined to be pseudo-progression (N=1), and an MDT recommendation to opt for adjuvant durvalumab following a post-induction metabolic complete response in a patient who would have had to undergo resection of three vertebrae (N=1). Of 26 patients who underwent surgery, 1 was excluded from further analysis of clinical outcomes as the pathology specimen revealed metastasis from undifferentiated melanoma.

Patient characteristics are summarized in table 1. The median patient age was 64 years (range 43–73), most were current or former smokers (97%) and a majority presented with non-squamous histology (72%). Tumor PD-L1 expression was 50% or more in 63% of patients. A comparison of baseline characteristics of patients with and without pCR is shown in online supplemental table 3.

**Table 1:** Baseline patient characteristics

Factor	Category	n=30 (%)
Age (median, range)		64 (43–73)
BMI (median, range)		27 (19–33)
Sex	Male	14 (47)
	Female	16 (53)
Smoking status*	Never	1 (3)
	Former	12 (40)
	Current	17 (57)
AID	Yes	2 (7)
	No	28 (93)
ECOG	0	18 (60)
	1	12 (40)
T-stage	T3	11 (37)
	T4	19 (63)
N-stage	N0	18 (60)
	N1	9 (30)
	N2	3 (10)
Histology	Non-squamous	21 (70)
	Squamous	8 (27)
	Melanoma lung metastasis	1 (3)
PD-L1	50% or above	12 (40)
	1–49%	3 (10)
	Less than 1%	4 (13)
	Unknown	11 (37)
AGA	0	28 (93)
	1	2 (7)
Chemotherapy	1	3 (10)
	2	26 (87)
	3	1 (3)
Immunotherapy	1	4 (13)
	2	26 (87)
Thoracic radiotherapy dose	60 Gy	3 (10)
	50 Gy	25 (83)
	Less than 50 Gy	2 (7)

* (*) Never smoker =≤100 cigarettes in a life-time, former=stopped smoking for >1 year, current=active smoker or has stopped for <1 year.

AGAactionable genomic alterationAIDautoimmune disease in medical historyBMIbody mass indexECOGperformance status scale according to the Eastern Cooperative Oncology GroupPD-L1programmed death ligand-1

### Adverse events and surgical outcomes

Adverse events were scored in all 30 patients who commenced induction therapy. All patients experienced treatment-emergent adverse events (TEAEs), with 25 (83%) patients experiencing grade 3 or higher toxicity (online supplemental table 4). Table 2 summarizes treatment-related adverse events (TRAEs) recorded in at least three (10%) patients (a full list is provided in online supplemental table 5). One or more grade 3 immune-related adverse events were recorded in seven (23%) patients, leading to discontinuation of NIVO during the second cycle in 10% of patients. Two patients died during the study: one due to COVID-19 and another due to pneumonitis-induced respiratory failure 96 days after surgery. The latter developed rapidly progressive interstitial lung disease approximately 100 days after the last fraction of radiotherapy, for which no infectious cause was identified. The fatal event was scored as a late treatment-related immune-related adverse event.

**Table 2:** Treatment-related adverse events

TRAEs		Any graden (%)	Grade 1–2n	Grade 3–4n	Grade 5n
	Any	30 (100)	30	21	1
	Dermatitis/rash	24 (80)	22	2	0
	Appetite loss/nausea/vomiting	21 (70)	19	2	0
	Anemia	21 (70)	19	2	0
	Fatigue	18 (60)	17	1	0
	Transaminitis/hepatitis	17 (57)	13	4	0
	Leukopenia	17 (57)	10	7	0
	Esophagitis	16 (53)	15	1	0
	Electrolyte disorders	16 (53)	15	1	0
	Constipation/diarrhea	16 (53)	16	0	0
	Thrombocytopenia	14 (47)	9	5	0
	Pain	13 (43)	13	0	0
	Alk Phos increased	11 (37)	11	0	0
	Thyroid disorders	10 (33)	10	0	0
	Dyspnea	10 (33)	7	3	0
	Cough	10 (33)	10	0	0
	GGT increased	8 (27)	6	2	0
	Fever	6 (20)	6	0	0
	Nervous disorders	5 (17)	5	0	0
	Lymphopenia	5 (17)	4	1	0
	LDH increased	5 (17)	5	0	0
	Pneumonitis	4 (13)	2	1	1
	Dry skin	4 (13)	4	0	0
	Insomnia	3 (10)	3	0	0
	Infusion-related reaction	3 (10)	3	0	0
	CRP increased	3 (10)	3	0	0
**irAEs**					
	Any	20 (67)	16	6	1
	Thyroid disorders	10 (33)	10	0	0
	Dermatitis/rash	9 (30)	7	2	0
	Pneumonitis	3 (10)	2	0	1
	Transaminitis/hepatitis	2 (7)	0	2	0
	Pericarditis	1 (3)	1	0	0
	Pancreatitis	1 (3)	0	1	0
	Myositis	1 (3)	0	1	0
	Allergic reaction	1 (3)	1	0	0

TRAEs monitored in all 30 patients commencing with induction therapy, and occurring in 10% or more of patients, in the period from the start of therapy until 90 days post-surgery or if no surgery was performed until 180 days after the start of therapy, are shown in the top part. In the bottom part, irAEs are shown.

CRPC-reactive proteinGGTgamma glutamyl transferaseirAEimmune-related adverse eventsLDHlactate dehydrogenaseTRAEtreatment-related adverse events

A total of 26 out of 30 patients underwent surgery, resulting in an 87% resection rate in the ITT population. The median time from the last radiotherapy fraction to surgical resection was 42 days (range 35–63 days). TRAE’s did not lead to failures to undergo surgery. A total of 25 lobectomies (96%) and 1 pneumonectomy (4%) were performed, all with a systematic nodal dissection. A pathological complete (R0) resection was achieved in all patients. Surgical complications occurred in 16/25 patients, scored as Clavien-Dindo grade 2 complications in 12 patients, grade 3a complications in 3 patients, and grade 3b complications in 2 patients (20% grade 3a-b complications). No 30-day or 90-day mortality was observed. A pneumo-pleural fistula developed in one patient, which resolved spontaneously on antibiotics.

### Pathological responses

**Figure 2:**
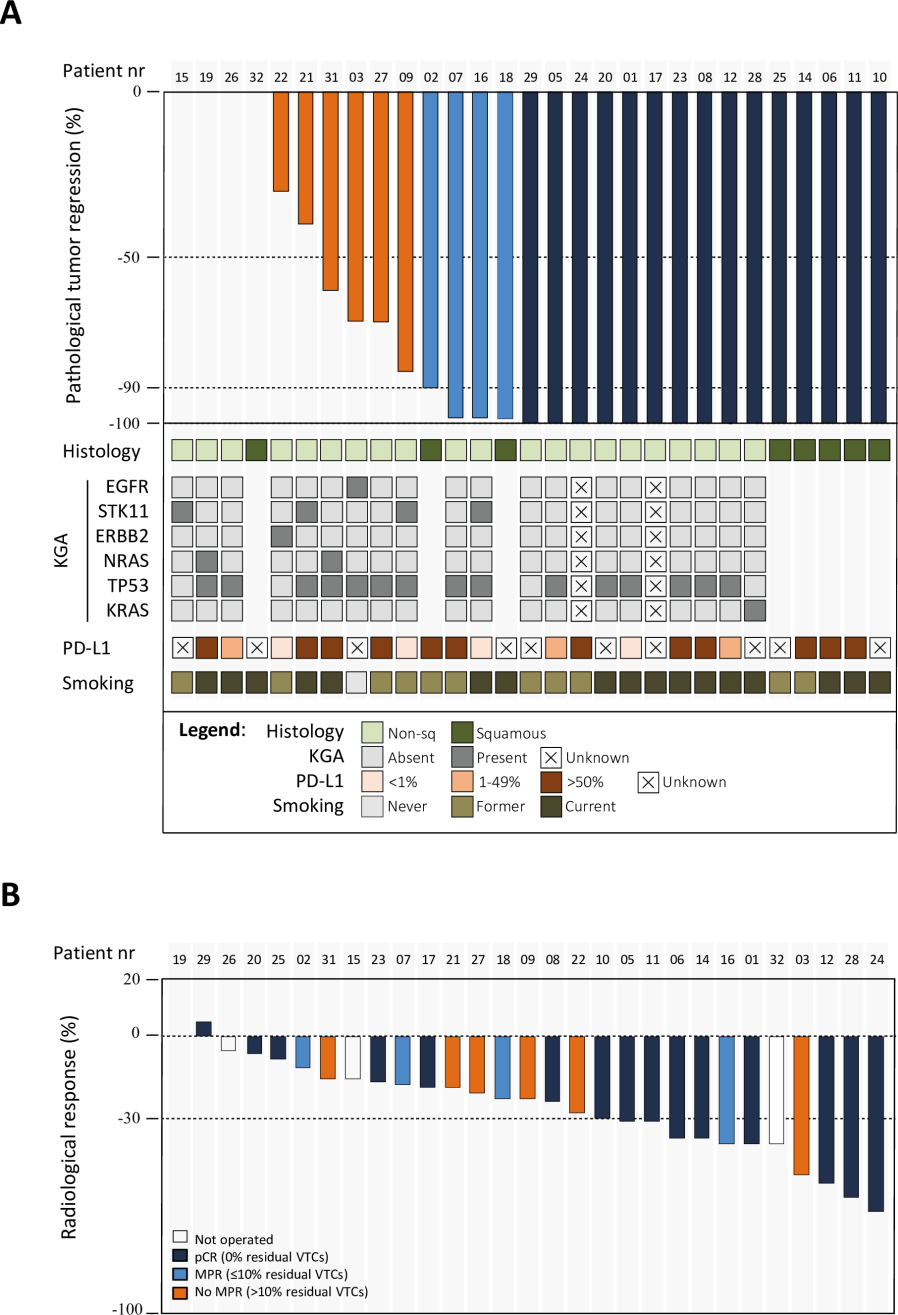
(**A**) The waterfall plot for pathological response (top row), shows that 15 (60%) out of 25 operated patients with NSCLC (excluding the patient with melanoma) achieved a pCR (dark blue bars), another 4/25 (16%) achieved an MPR (light blue bars), the remaining 6 (24%) patients had more than 10% residual viable tumor cells left (orange bars). In operated non-squamous cell lung cancers, genomic alterations associated with resistance to immunotherapy (EGFR, ERBB2, STK11 and NRAS) were seen in eight patients, all of which had no pCR. An STK11 mutation was seen in the only patient who developed PD on induction therapy (ie, patient 15). TP53 was seen in 81% (13/16) of operated patients where genomic analysis was performed, while only one tested patient had a KRAS mutation. Only 28% (7/25) of operated patients with NSCLC had squamous cell histology (dark green). All, except for one patient, were current or former smokers. The only never-smoker (patient 03) had an EGFR exon 21 (L858R) mutation, which was found post-surgery. High tumor PD-L1 expression rates were observed in 56% (14/25) of patients with resected NSCLC (bottom row). (**B**) Radiological tumor responses to the induction therapy, that is, change in the sum of longest diameters according to RECIST V.1.1, are shown in the patients with NSCLC. Partial response was seen in 11 patients and stable disease in 16 patients. In patient 32, pseudo-progression with new lung nodules were seen, while the primary tumor shrunk. In patient 15, PD was seen due to the appearance of new pleural lesions on induction therapy, while the treated baseline tumor lesions shrunk. Patient 19 did not complete induction therapy and could therefore not be evaluated. Patients with pCR (dark blue), MPR (light blue) and no MPR (orange) can be seen among the patients with radiological partial response and stable disease. KGA, key genomic alterations; MPR, major pathological response; NSCLC, non-small cell lung cancer; pCR, pathological complete response; PD, progressive disease; PD-L1, programmed death ligand-1; VTC, viable tumor cell.

Of the 26 patients who underwent a resection, 7 patients, including the patient with pulmonary melanoma, had more than 10% residual viable tumor cells. From the remaining patients, 15 (58%; 95% CI: 37% to 77%) achieved a pCR, and the other four had an MPR (73%; 95% CI: 52% to 88%). For the 25 operated patients with NSCLC (excluding the single patient with melanoma), the pCR and MPR rates were 60% (95% CI: 39% to 79%) and 76% (95% CI: 55% to 91%). For the ITT, these were 50% (95% CI: 31% to 69%) and 63% (95% CI: 44% to 80%), respectively (figure 2).

Baseline tumor PD-L1 expression of 50% or above was seen in 63% of patients, however, the presence of high PD-L1 expression was not statistically different among patient groups based on the presence of a pCR or MPR. Online supplemental table 6 shows the full list of PD-L1 results.

Six out of the eight patients with non-squamous NSCLC who underwent surgery but did not achieve pCR were found to have genomic alterations known to be associated with resistance to immunotherapy (figure 2A).^16^,^19^ The single patient who developed disease progression on induction therapy had an STK11 mutation. Detailed results from the molecular analysis is shown in online supplemental table 7.

### Radiological and ^18^F-FDG responses

Figure 2B summarizes the depth of radiological responses on CT scan to induction therapy, that is, change in the sum of longest diameters according to Response Evaluation Criteria in Solid Tumors (RECIST) V.1.1, in the ITT NSCLC population. No patient achieved a radiological complete response. Partial responses (PR) were seen in 11 (37%) patients, and stable disease in 16 (53%) patients (SD). pCR was not different between patients with PR and those without (p=0.096). Two patients developed new lesions on induction therapy while the treated baseline tumor lesion shrunk. Of these, one patient developed pleural lesions that were scored as progressive disease (PD), and one patient developed bilateral new pulmonary lesions during therapy, which were initially scored as PD, but subsequently regressed spontaneously, and were scored as pseudo-progression.

**Figure 3:**
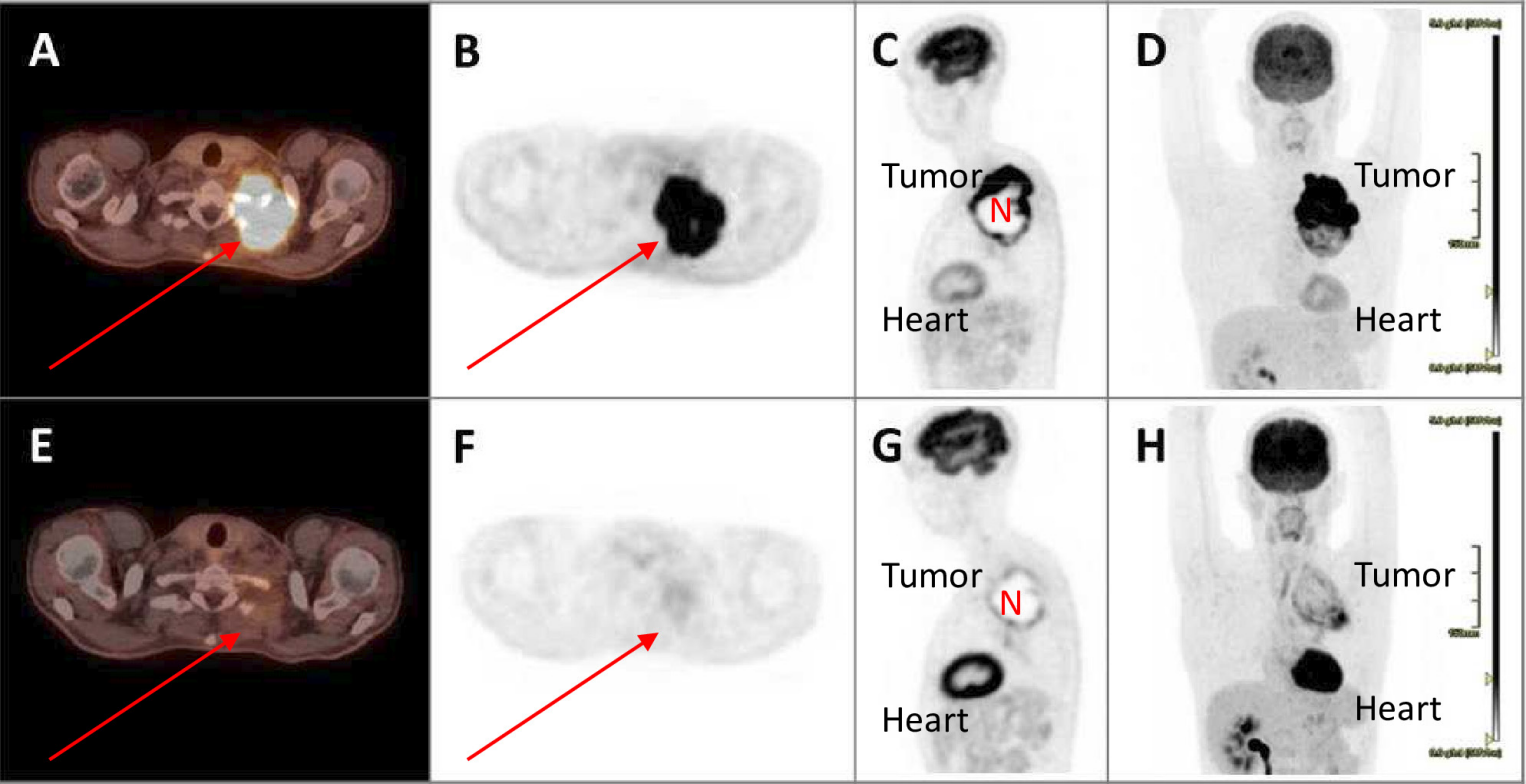
The FDG PET/CT images of an adult patient (23), before (top row) and after (bottom row) induction chemo-immuno-radiotherapy are shown, using fused PET/CT images (**A,E**) and CT-attenuation corrected PET images of axial (**B,F**), sagittal (**C,G**) and coronal view (**D,H**) reconstructions. This patient had a tumor in the left upper lobe with, at baseline, ingrowth in the thoracic wall and adjacent structures such as the musculature and left ribs (red arrows). The tumor had a necrotic center (N), and there was no apparent mediastinal lymph node involvement. CTAC, CT-attenuation correction; FDG, fluorodeoxyglucose; PET, positron emission tomography; RECIST, Response Evaluation Criteria in Solid Tumors.

Figure 3 illustrates the radiological and metabolic responses observed in an adult patient with an adenocarcinoma in the left upper lobe invading adjacent thoracic vertebra and extrathoracic tissues. The post-surgical pathology specimen revealed a pCR. The patient was discharged 11 days after surgery, and remains free of disease 16 months after resection, without a need for pain medication.

### Survival outcomes

Per August 2023, at a median follow-up time of 25.8 months (range 1.0–42.3), in 8 out of 30 patients, disease recurrence was seen, and 7 patients died. These initial survival outcomes, including their respective events, are shown in online supplemental figure 6 and table 8.

### Immunological responses

As resected tumor tissues were mostly necrotic, these were found to be unsuitable for substantive analysis.

### Peripheral blood immune monitoring

**Figure 4:**
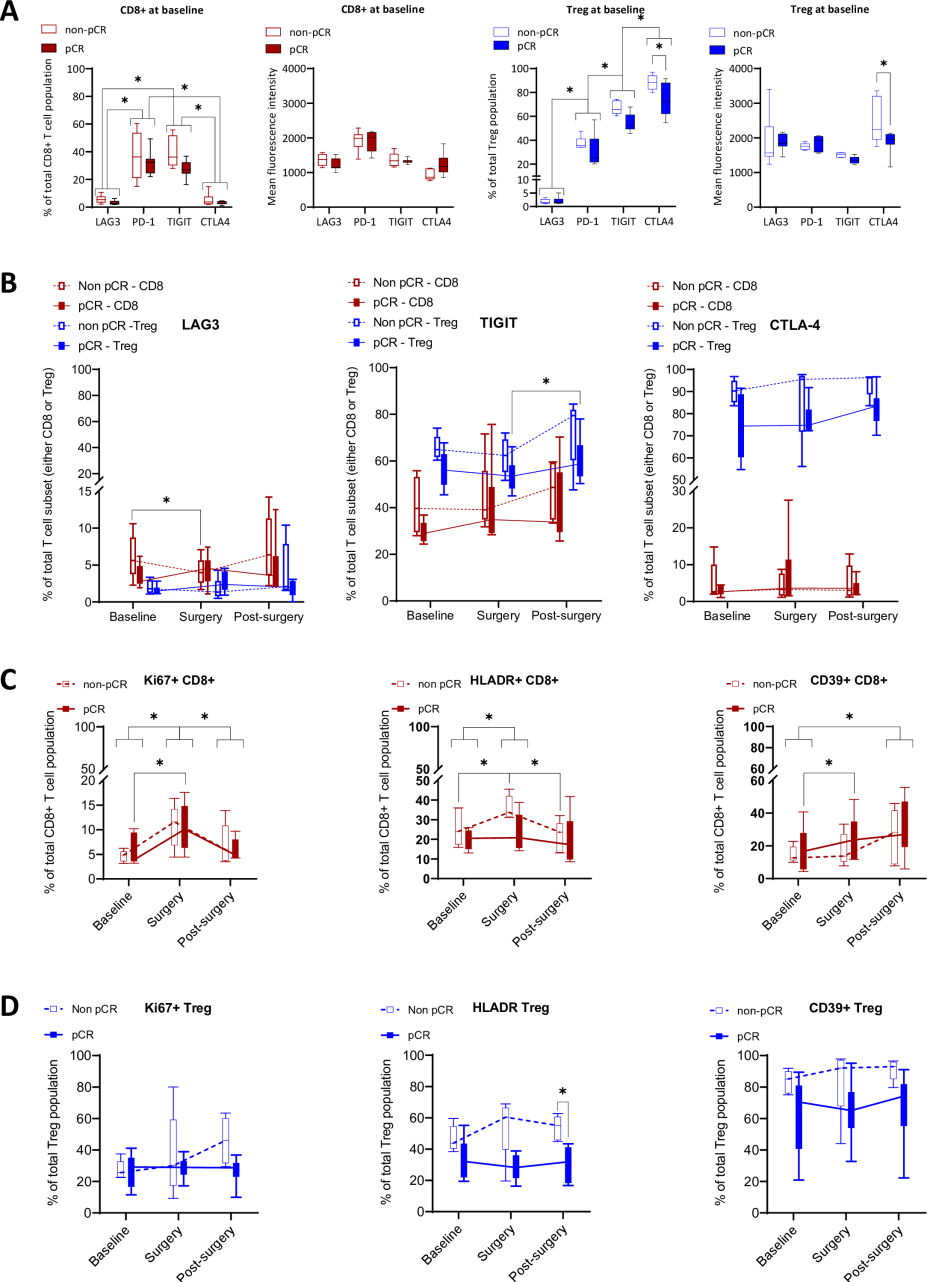
Peripheral blood T-cell subset analysis and its correlation with pathological response. The tests used and the exact p values are shown in the (online supplemental data chapter 6 figures 2–5), here, asterisks indicate significant differences (p<0.05). (**A**) Immune checkpoint expression in CD8+T cells (red, first two panels) and regulatory T cells (blue, last two panels) from 13 patients at baseline. Data is shown as a “percentage of the T cell population”, which indicates the proportion of cells expressing a marker within the total CD8+T cell or Treg population, as well as the “mean fluorescence intensity” for each T-cell subset. Solid symbols denote patients with pCR (N=7) and hollow symbols denote those without pCR (N=6). Medians and ranges are displayed for each immune checkpoint. (**B**) Longitudinal expression of LAG3, TIGIT, and CTLA-4 in CD8+T cells (red) and Tregs (blue) for 11 patients at baseline, surgery, and 12 weeks post-surgery as a percentage of the total population of T-cell subsets (either CD8 or Treg). Solid symbols represent patients with pCR (N=6) and hollow symbols represent those without pCR (N=5). (**C–D**) Markers for proliferation (Ki67), activation (HLA-DR), and tumor association (CD39) in CD8+T cells (figure 4C) and Tregs (figure 4D) are shown for 11 patients at baseline, surgery, and 12 weeks post-surgery as a percentage of the total population of T-cell subsets (either CD8 or Treg). Solid symbols denote patients with pCR (N=6) and hollow symbols represent those without pCR (N=5). CTLA-4, cytotoxic T-lymphocyte associated protein 4; LAG3, Lymphocyte Activation Gene 3; pCR, pathological complete response; TIGIT, T cell immunoreceptor with immunoglobulin and ITIM domain; Treg, regulatory T cells.

Figure 4A displays the baseline immune checkpoint expression as a percentage of the CD8+T cell and Treg populations. Alongside this, the mean fluorescence intensity is presented, which aligns well with the percentages, highlighting relative differences in immune checkpoint expressions. At baseline, CD8+T cells predominantly expressed PD-1 and T cell immunoreceptor with immunoglobulin and ITIM domain (TIGIT) over Lymphocyte Activation Gene 3 (LAG3) and CTLA-4. In Tregs, LAG3 had the least expression, followed by PD-1, then TIGIT, with CTLA-4 being the highest. Among patients without pCR, most immune checkpoint expressions were similar or higher (non-significant) than in those with pCR, both in CD8+T cells as well as Tregs. Notably, only CTLA-4 expression in Tregs was significantly higher in patients without pCR.

Reliable post-induction PD-1 measurements were hindered by interference from therapeutic anti-PD-1 with the Fluorescence-activated cell sorting (FACS) antibody. This was not a problem for intracellularly measured CTLA-4. Figure 4B presents longitudinal immune checkpoint changes. LAG3 was consistently low, with a slight drop in CD8+T cells of non-pCR patients from baseline to surgery. TIGIT was stable initially but increased post-surgery, significantly so in pCR Tregs. While CTLA-4 expression in CD8+T cells was low at baseline and stayed low, its expression in Tregs was notably higher and showed an increasing trend over time. Notably, CTLA-4 expression was significantly higher in patients without pCR compared with those with pCR, both at baseline and overall (p=0.0197).

Figure 4C–D displays Ki67+, HLA-DR+, and CD39+expression on CD8+T cells and Tregs at baseline, surgery, and 12 weeks post-surgery. Ki67 indicates cell proliferation, HLA-DR signals T-cell activation, and CD39 suggests tumor-reactivity.^20^ Post-induction, CD8+T cells showed increased Ki67 and HLA-DR levels, both reverting to baseline post-surgery. CD39+CD8+ T cells rose post-induction and remained elevated 12 weeks post-surgery, especially in patients with pCR (p=0.0476). Ki67+Treg levels remained stable during induction therapy and post-surgery. However, upregulation of HLA-DR was observed in patients who failed to achieve a pCR, and CD39+Treg frequencies showed a trend towards an increase throughout treatment and follow-up (non-significant).

Further testing details are in the supplementary data, namely online supplemental figures 2–5 and the PBMC test comparison tables.

## Discussion

To the best of our knowledge, the INCREASE study is the first to investigate the safety and efficacy of adding dual immunotherapy, using a PD-1 and a CTLA-4 blocker, to high-dose concurrent CRT in the neoadjuvant setting for locally advanced NSCLC. Our main findings were a pCR and MPR in 50% (15/30) and 63% (19/30) of the ITT population, and in 58% (15/26) and 73% (19/26) of patients who underwent surgery, respectively. The addition of dual immunotherapy to CRT substantially increased pCR rates above historical rates after CRT, which vary from 21% to 45%.^5 6 8^ A summary of pathological responses to various neoadjuvant regimens are provided in table 3.

**Table 3:** Comparison of pathological outcomes

Study	Population	Ph	N	PE	Treatment	Key results
Neoadjuvant immunotherapy monotherapy vs dual immunotherapy						
CANOPY-N NCT03968419	Stage IB-IIIA	II	88	MPR	Canakinumab vs pembrolizumab vs combined	Canakinumab: MPR 2.9%Pembrolizumab: MPR 11.1%Combined: MPR 17.1%
NeoCOAST NCT03794544	Stage I-IIIA	II	84	MPR	Durvalumab vs durvalumab+oleclumab vs durvalumab+monalizumab vs durvalumab+danvatirsen	Durvalumab: MPR 11.1%Oleclumab: MPR 19.0%Monalizumab: MPR 30.0%Danvatirsen: MPR 31.3%
NEOSTAR NCT03158129	Stage I-IIIA	II	44	MPR	Nivolumab vs nivolumab+ipilimumab	Nivolumab: MPR 22%Nivo+ipilimumab: MPR 38%
Neoadjuvant chemotherapy vs chemoimmunotherapy						
TD-FOREKNOW NCT04338620	Stage IIIA-IIIB	II	88	pCR	CT+camrelizumab vs CT	CT+camrelizumab: pCR 32.6%CT: pCR 8.9%
CheckMate 816 NCT02998528	Stage IB-IIIA	III	358	EFS, pCR	CT+nivolumab vs CT	CT+nivolumab: pCR 24.0%, MPR 36.9%CT: pCR 2.2%, MPR 8.9%
NADIM II NCT03838159	Stage IIIA-IIIB	II	86	pCR	CT+nivolumab vs CT	CT+nivolumab: pCR 37%CT: pCR 7%
AEGEAN NCT03800134	Stage II-IIIB	III	740	EFS, pCR	CT+durvalumab vs CT	Durvalumab: pCR 17.2%, MPR 33.3%CT: pCR 4.3%, MPR 12.3%
CheckMate 77T NCT04025879	Stage II-IIIB	III	461	EFS	CT+nivolumab vs CT	Nivolumab: pCR 25.3%, MPR 35.4%CT: pCR 4.7%, MPR 12.1%
KEYNOTE-671 NCT03425643	Stage II-IIIB	III	797	EFS, OS	CT+pembrolizumab vs CT	Pembrolizumab: pCR 18.1%, MPR 30.2%CT: pCR 4.0%, MPR 11.0%
Neotorch NCT04158440	Stage II-III	III	404	EFS, MPR	CT+toripalimab vs CT	Toripalimab: MPR 48.5%, pCR 24.8%CT: MPR 8.4%, pCR 1.0%
RATIONALE-315 NCT04379635	Stage II-IIIA	III	453	EFS, MPR	CT+tislelizumab vsCT	Tislelizumab: MPR 56.2%, pCR 40.7%CT: MPR 15.0%, pCR 5.7%
Neoadjuvant chemoradiotherapy						
Albain *et al* PMID19632716	Stage IIIA(N2)	III	396	OS	CT/RT/S vs CT/RT	pCR: 18%
Epithor PMID37156211	T3-4 tumors	RS	688	OS	S vs CT+S vs CT/RT/S	CRT: pCR: 18.5%CT: pCR: 6%
ESPATUE PMID26527789	Stage IIIA-B	III	246	OS	CT/RT/S vs CT/RT	pCR 33%
Cerfolio *et al* PMID19233668	Stage III	RS	216	DES	CT/RT/S	pCR 33%

CTchemotherapyDESdescriptive endpointsEFSevent-free survivalMPRmajor pathological responseOSoverall survivalpCRpathological complete responsePEprimary endpointsPhphaseRSretrospective studyRTradiotherapySsurgery

The rationale and results of our study closely align with those of the recently published platform NEOSTAR study, which reported increased pathological responses using the combination of NIVO and IPI with chemotherapy in resectable stage IB-IIIA NSCLC.^21^ The study successfully met its primary endpoint of MPR, with 32.1% (7/22, 80% 95% CI: 18.7% to 43.1%) in the NIVO+CT arm and 50% (11/22, 80% 95% CI: 34.6% to 61.1%) in the IPI+NIVO+CT arm.

While MPR or pCR are not a survival endpoint, following neoadjuvant therapy, they have been found to potentially predict DFS and OS, regardless of the neoadjuvant treatment modality. Pataer *et al* showed that in patients with resectable NSCLC, MPR following neoadjuvant chemotherapy was associated with a longer OS.^22^ Analysis of our previous institutional outcomes for sulcus superior tumors treated with CRT and surgery identified pCR as predictive for improved 5-year OS, with an HR of 0.27 (95% CI: 0.15 to 0.50; p<0·001).^6^ In the phase-III CheckMate-816 study, investigating neoadjuvant chemotherapy with or without NIVO in resectable NSCLC, the residual percentages of vital tumor cells also predicted for event-free survival at 2 years in the chemo-NIVO arm (area under the curve (AUC)=0.74).^23^ Of note, although the high PD-L1 expression levels that were observed in 63% of patients in this study might be expected to influence pathological responses, pCR were observed in tumors expressing high, low, and absent PD-L1 expression levels. This is also in line with the observations in the IPI-NIVO-CT arm of the NEOSTAR platform study, but also CheckMate-9LA and CheckMate-227, where the efficacy of IPI-NIVO-CT was irrespective of tumor PD-L1 expression levels.^21 24 25^

Despite excluding patients with an identified targetable oncogenic driver before study inclusion, surgical specimens revealed the presence of such drivers. One patient with an EGFR exon 21 (L858R) mutation had 30% residual viable tumor cells in the resected tumor, and another patient with an ERBB2 mutation had 70% residual viable tumor cells post-induction therapy. In addition, four patients with STK-11 mutations were identified, one of whom had a pCR and one developed disease progression post-induction therapy. These findings suggest that genomic features associated with immunotherapy resistance may adversely influence the efficacy of the IPI-NIVO-CRT combination. However, caution is warranted in view of the limited number of patients.

As reported previously for neoadjuvant NSCLC studies, radiological responses observed to this induction therapy did not correlate with pCR.^26 27^ Indeed, INCREASE patients with a pCR showed a best radiological response of PR in 53%, and SD in 47%. These findings highlight a need for improving response evaluation to neoadjuvant therapies.

In the ITT population, 83% suffered grade ≥3 TEAEs and 73% encountered TRAEs. However, these toxicities did not prevent surgery, and toxicity rates aligned with CRT studies like the PROCLAIM study, where 64% and 76.8% grade 3–4 toxicities were reported.^28^ The current study identified immune-related events, with 23% being grade ≥3, including one fatal pneumonitis. A recent study combining CRT with IPI and NIVO reported a 63% pulmonary toxicity rate.^29^ Our lower pneumonitis rate might be the result of resecting irradiated lung tissues and using lower radiation doses (50 Gy in 86% of patients). The influence of unresected irradiated lung tissue on high-grade pulmonary toxicity needs further exploration.^30^

The remarkable improvement in pathological tumor response observed with the IPI/NIVO and CRT combination might be a result of enhanced immunogenic cell death, caused by the proliferation and activation of effector T cells and the suppression of Tregs. Indeed, PBMC analysis revealed a noticeable post-induction surge in proliferative and activated CD8+effector T cells, as evidenced by increased Ki67+and HLA-DR+populations. However, this surge returned to baseline levels post-surgery. Intriguingly, CD39, a marker linked to tumor-reactivity, demonstrated a post-induction rise that was sustained for up to 12 weeks post-surgery, suggesting the continuing presence of tumor-reactive effector T cells. Meanwhile, suppressive Tregs did not proliferate, and interestingly, their activation was notably less in patients achieving a pCR.

Baseline data revealed that CD8+T cells expressed CTLA-4 less than other immune checkpoints, whereas Tregs had a markedly high CTLA-4 expression. Also, CTLA-4 expression in Tregs was even higher in patients without a pCR compared with those achieving pCR. When observing longitudinal changes, CTLA-4’s expression pattern stood out. While its levels in CD8+T cells remained consistently low, Tregs exhibited a pronounced and increasing expression over time, suggesting that IPI’s blockade of CTLA-4 could be of considerable therapeutic importance in reducing the immune suppressive effects of Tregs.

These findings align well with the results of the NEOSTAR study, particularly the IPI-NIVO-chemotherapy arm, showing increased CD3+CD8+ tumor-infiltrating T cells and heightened antigen-activated T cells post-therapy.^21^ However, NEOSTAR also showed some interesting leads into other cell lines such as B-cell abundance and its inverse correlation with viable tumor cells, suggesting an association with immunotherapeutic response. Additionally, CXCL9+tumor-associated macrophages exhibited a functional role in response to immune checkpoint therapy, indicating enhanced immune activation, effector memory, cytotoxic function, and reduced immunosuppressive cell subsets in treated tumors.

Some limitations of this exploratory single-center, single-arm study are acknowledged. Despite a significant increase in pathological response, namely pCR rates, it is unclear whether pCR alone could function as a reliable surrogate endpoint, even though data from large neoadjuvant studies is suggestive of this.^23 31 32^

A substantial number of TRAEs was observed, which, although manageable, suggests that critical assessment of patient selection, drug dosage and therapy de-escalation all deserve further study. Other trials are currently investigating the immune-modulatory effects of different fractionation regimens of radiotherapy to enhance the effects of neoadjuvant PD-L1 blockade after neoadjuvant chemotherapy in patients with resectable stage III (N2) NSCLC.

The expected outcome of this trial was a 60% pCR rate, this was not achieved in the ITT and resected populations, the operated NSCLC population did reach this rate, with 15 out of 25 patients achieving pCR (60%). However, the initial sample size calculation was powered at 90%, but when considering an 80% power, both the ITT and resected populations meet the criteria. It is important to emphasize that this was an exploratory single-arm phase 2 study designed to assess the impact of adding dual immunotherapy to CRT rather than strictly meeting the 60% pCR benchmark.

The postoperative resection specimens, collected on average 6 weeks post-radiotherapy, were predominantly necrotic. This timing likely led to the loss of tumor immune dynamics and profiles typical of dual immunotherapy with CRT within the resection specimens. Consequently, the remaining tissue primarily consisted of mainly necrotic tumor beds, unsuitable for meaningful translational analysis.

This regimen is unlikely to be adopted in routine clinical practice due to its complexity and the necessity for it to be conducted in highly specialized centers that are proficient in complex surgeries post-CRT and managing severe toxicities. Despite this, the regimen fills a critical knowledge gap and provides valuable insights into the multimodality treatment landscape. There is an ongoing need to enhance treatment outcomes while minimizing toxicity and patient burden. The ultimate goal within the scientific community is to refine the dose, sequence, and frequency of these multimodality treatment components, including various immunotherapies combined with chemotherapy, radiotherapy, and surgery, to achieve optimal local and distant control with the least amount of adverse events.

## Conclusion

This study revealed that the use of neoadjuvant dual immunotherapy with CRT in T3-4N0–2 NSCLC resulted in enhanced pCR and MPR with acceptable surgical morbidity. Patients with pCR demonstrated higher proliferation and activation rates of CD8^+^ T cells and lower rates in Tregs, these observations warrant further investigation.

## supplementary material

10.1136/jitc-2024-009799 online supplemental figure 1

## Data Availability

Data are available upon reasonable request.

## References

[1] Ferlay J, Colombet M, Soerjomataram I (2019). Estimating the global cancer incidence and mortality in 2018: GLOBOCAN sources and methods. Int J Cancer.

[2] Postmus PE, Kerr KM, Oudkerk M (2017). Early and locally advanced non-small-cell lung cancer (NSCLC): ESMO Clinical Practice Guidelines for diagnosis, treatment and follow-up. Ann Oncol.

[3] Daly ME, Singh N, Ismaila N (2022). Management of Stage III Non-Small-Cell Lung Cancer: ASCO Guideline. J Clin Oncol.

[4] Van Meerbeeck JP, Van Schil PEY, Senan S (2007). Reply: Randomized controlled trial of resection versus radiotherapy after induction chemotherapy in stage IIIA-N2 non-small cell lung cancer. J Thorac Oncol.

[5] Albain KS, Swann RS, Rusch VW (2009). Radiotherapy plus chemotherapy with or without surgical resection for stage III non-small-cell lung cancer: a phase III randomised controlled trial. Lancet.

[6] Ünal S, Winkelman JA, Heineman DJ (2023). Long-Term Outcomes After Chemoradiotherapy and Surgery for Superior Sulcus Tumors. JTO Clin Res Rep.

[7] Tricard J, Filaire M, Vergé R (2023). Multimodality therapy for lung cancer invading the chest wall: A study of the French EPITHOR database. Lung Cancer (Auckl).

[8] Eberhardt WEE, Pöttgen C, Gauler TC (2015). Phase III Study of Surgery Versus Definitive Concurrent Chemoradiotherapy Boost in Patients With Resectable Stage IIIA(N2) and Selected IIIB Non-Small-Cell Lung Cancer After Induction Chemotherapy and Concurrent Chemoradiotherapy (ESPATUE). J Clin Oncol.

[9] Dickhoff C, Hartemink KJ, Kooij J (2017). Is the routine use of trimodality therapy for selected patients with non-small cell lung cancer supported by long-term clinical outcomes?. Ann Oncol.

[10] Cerfolio RMJ, Bryant AS, Jones VL (2009). Pulmonary resection after concurrent chemotherapy and high dose (60Gy) radiation for non-small cell lung cancer is safe and may provide increased survival☆. Eur J Cardiothorac Surg.

[11] Cascone T, William WN, Weissferdt A (2021). Neoadjuvant nivolumab or nivolumab plus ipilimumab in operable non-small cell lung cancer: the phase 2 randomized NEOSTAR trial. Nat Med.

[12] Formenti SC, Rudqvist N-P, Golden E (2018). Radiotherapy induces responses of lung cancer to CTLA-4 blockade. Nat Med.

[13] Goldstraw P, Chansky K, Crowley J (2016). The IASLC Lung Cancer Staging Project: Proposals for Revision of the TNM Stage Groupings in the Forthcoming (Eighth) Edition of the TNM Classification for Lung Cancer. J Thorac Oncol.

[14] Dickhoff C, Senan S, Schneiders FL (2020). Ipilimumab plus nivolumab and chemoradiotherapy followed by surgery in patients with resectable and borderline resectable T3-4N0-1 non-small cell lung cancer: the INCREASE trial. BMC Cancer.

[15] Cottrell TR, Thompson ED, Forde PM (2018). Pathologic features of response to neoadjuvant anti-PD-1 in resected non-small-cell lung carcinoma: a proposal for quantitative immune-related pathologic response criteria (irPRC). Ann Oncol.

[16] Hastings K, Yu HA, Wei W (2019). EGFR mutation subtypes and response to immune checkpoint blockade treatment in non-small-cell lung cancer. Ann Oncol.

[17] Skoulidis F, Goldberg ME, Greenawalt DM (2018). STK11/LKB1 mutations and PD-1 inhibitor resistance in KRAS-mutant lung adenocarcinoma. Cancer Discov.

[18] Mazieres J, Drilon A, Lusque A (2019). Immune checkpoint inhibitors for patients with advanced lung cancer and oncogenic driver alterations: results from the IMMUNOTARGET registry. Ann Oncol.

[19] Zhou L, Wang X, Chi Z (2021). Association of NRAS Mutation With Clinical Outcomes of Anti-PD-1 Monotherapy in Advanced Melanoma: A Pooled Analysis of Four Asian Clinical Trials. Front Immunol.

[20] Kortekaas KE, Santegoets SJ, Sturm G (2020). CD39 Identifies the CD4^+^ Tumor-Specific T-cell Population in Human Cancer. Cancer Immunol Res.

[21] Cascone T, Leung CH, Weissferdt A (2023). Neoadjuvant chemotherapy plus nivolumab with or without ipilimumab in operable non-small cell lung cancer: the phase 2 platform NEOSTAR trial. Nat Med.

[22] Pataer A, Weissferdt A, Correa AM (2022). Major Pathologic Response and Prognostic Score Predict Survival in Patients With Lung Cancer Receiving Neoadjuvant Chemotherapy. JTO Clin Res Rep.

[23] Provencio-Pulla M, Spicer J, Taube JM (2022). Neoadjuvant nivolumab (NIVO) + platinum-doublet chemotherapy (chemo) versus chemo for resectable (IB–IIIA) non-small cell lung cancer (NSCLC): Association of pathological regression with event-free survival (EFS) in CheckMate 816. J C O.

[24] Paz-Ares L, Ciuleanu T-E, Cobo M (2021). First-line nivolumab plus ipilimumab combined with two cycles of chemotherapy in patients with non-small-cell lung cancer (CheckMate 9LA): an international, randomised, open-label, phase 3 trial. Lancet Oncol.

[25] Brahmer JR, Lee J-S, Ciuleanu T-E (2023). Five-Year Survival Outcomes With Nivolumab Plus Ipilimumab Versus Chemotherapy as First-Line Treatment for Metastatic Non–Small-Cell Lung Cancer in CheckMate 227. JCO.

[26] Cascone T, William WN, Weissferdt A (2019). Neoadjuvant nivolumab (N) or nivolumab plus ipilimumab (NI) for resectable non-small cell lung cancer (NSCLC): Clinical and correlative results from the NEOSTAR study. J C O.

[27] William WN, Pataer A, Kalhor N (2013). Computed tomography RECIST assessment of histopathologic response and prediction of survival in patients with resectable non-small-cell lung cancer after neoadjuvant chemotherapy. J Thorac Oncol.

[28] Senan S, Brade A, Wang L-H (2016). PROCLAIM: Randomized Phase III Trial of Pemetrexed-Cisplatin or Etoposide-Cisplatin Plus Thoracic Radiation Therapy Followed by Consolidation Chemotherapy in Locally Advanced Nonsquamous Non-Small-Cell Lung Cancer. J Clin Oncol.

[29] Liveringhouse CL, Latifi K, Asous AG (2023). Dose-Limiting Pulmonary Toxicity in a Phase 1/2 Study of Radiation and Chemotherapy with Ipilimumab Followed by Nivolumab for Patients With Stage 3 Unresectable Non-Small Cell Lung Cancer. Int J Radiat Oncol Biol Phys.

[30] Wu TC, Stube A, Felix C (2023). Safety and Efficacy Results From iSABR, a Phase 1 Study of Stereotactic ABlative Radiotherapy in Combination With Durvalumab for Early-Stage Medically Inoperable Non-Small Cell Lung Cancer. Int J Radiat Oncol Biol Phys.

[31] Felip E, Altorki N, Zhou C (2021). Adjuvant atezolizumab after adjuvant chemotherapy in resected stage IB–IIIA non-small-cell lung cancer (IMpower010): a randomised, multicentre, open-label, phase 3 trial. The Lancet.

[32] Wakelee HA, Altorki NK, Zhou C (2021). IMpower010: Primary results of a phase III global study of atezolizumab versus best supportive care after adjuvant chemotherapy in resected stage IB-IIIA non-small cell lung cancer (NSCLC). J C O.

