# Supplementary material for: Single-arm trial of neoadjuvant ipilimumab plus nivolumab with chemoradiotherapy in patients with resectable and borderline resectable lung cancer: the INCREASE study
Source: J Immunother Cancer. 2024 Sep 30;12(9):e009799. doi: 10.1136/jitc-2024-009799 (PMC11448277; doi:10.1136/jitc-2024-009799)
Supplement: online supplemental figure 1 [file jitc-12-9-s001.docx]

Supplementary Materials

# Diagnostic and treatment procedures

Patients were treated with CRT and surgery in accordance with ESMO guidelines. Chemotherapy using platinum-doublets was administered in 3-weekly cycles, using carboplatin (AUC 5 IV) on day 1 of each cycle and pemetrexed (500 mg/m2 IV) on day 1 of each cycle in non-squamous cell tumors. Squamous cell tumors were treated with carboplatin (AUC 5 IV) on day 1 of each cycle and etoposide (100 mg/m2 IV on days 1, 2, and 3 of each cycle). Alternatively, etoposide could be administered orally at 200 mg/m2 per day, on days 2 and 3 in selected patients. A total of 2 cycles of a platinum-doublet were given. However, in exceptional cases, a single cycle of chemotherapy alone could be started prior to the concurrent IPI/NIVO/CRT treatment if the MDT decided that immediate initiation of systemic therapy was desirable, for example, due to delays in completing RT planning.

At 3 weeks post-CRT restaging included an FDG PET with a diagnostic CT scan and brain magnetic resonance imaging (MRI). Radiological assessments were based on RECIST v1.1 [15], SUVpeak (average SUV value of a 1mL sphere around the highest SUV pixel) and ^18^F-FDG PET. Surgery was performed 6 weeks after finishing CRT by thoracic surgeons experienced in complex pulmonary surgery, encompassing anatomical lung resections with en-bloc resection of invaded structures e.g. thoracic wall, diaphragm or pericardium, was performed together with systemic mediastinal nodal dissection.

# Definition of endpoints

## Primary endpoint definitions

Safety was assessed throughout the study. Adverse events (AEs) were evaluated according to the Common Terminology Criteria for Adverse Events version 5.0 (CTCAE v5.0, National Cancer Institute, NCI) and collected from the date of inclusion through 90 days following surgery, or 100 days following the last nivolumab application in case surgery was not performed. The definition of serious adverse events (SAE), rules for halting the study based on dose limiting toxicities, and rules for discontinuation or withdrawal were previously published [14]. Treatment-related adverse events (TRAEs) of special interest included hyperthyroidism, hypothyroidism, adrenal insufficiency, hypophysitis, skin reactions, myositis, nephritis, pyrexia, pancreatitis, diabetes, increased transaminases, colitis, diarrhea, nausea, pleural effusion, dyspnea, pneumonia, and pneumonitis. Surgical morbidity was recorded using the Clavien-Dindo classification for surgical complications. Also, those complications that led to delay or canceling of CRT or surgery were recorded.

The pCR was defined as the absence of any viable tumor cells (ypT0N0M0) in the surgical resection specimen. MPR was defined as 10% or less viable tumor cells in the surgical resection specimen [29,30].

## Secondary endpoint definitions

Response was evaluated radiologically using RECIST v1.1 criteria [15] and metabolically by means of ^18^F-FDG PET, using the semi-quantitative measurement SUVpeak (average SUV within a 1mL spherical volume around the highest pixel). In SUV calculation tumor uptake was normalized to bodyweight and the net injected dosage.

Time to local or distant recurrence was defined as the time from the start of induction therapy to the date of local or distant disease progression or death. Patients who are alive without disease progression were censored on the date of their last disease assessment. Overall survival (OS) was defined as the time from the start of induction therapy to death. Patients who were still alive were censored at the date of last contact.

## Exploratory endpoint, immune monitoring

This study aimed to characterize the changes in the tumor microenvironment (TME), TDLN, and peripheral blood mononuclear cells (PBMCs) prior to, and after, induction therapy and after surgery. The baseline tumor biopsy and the resection specimen were analyzed by routine hematoxylin and eosin (H&E) histology and immunohistochemistry (IHC) staining panels for assessment of PD-L1 expression and viable tumor cells. Multiparameter flow cytometry of immune effector subset rates and activation state in peripheral blood before, during and after IPI-NIVO-CRT was performed on a BD LSR Fortessa flow cytometer and obtained data were analyzed using FlowJo analysis software (BD Biosciences). Resected lymph nodes were formalin fixed and stained with CD8 and Ki67 (MIB1). Per lymph node, the paracortical zones with the most activated cells were considered hotspots. Activated cytotoxic T cells were manually counted by using the PaViewer, Digital Pathology Solutions (Philips) program.

# Tumor tissue, Peripheral Blood Mononuclear Cell analyses

Pathologic complete response was defined as the absence of any viable tumor cells (ypT0N0M0) in the surgical resection specimen (assessed in tumor and lymph nodes). MPR was defined as 10% or less viable tumor cells in the surgical resection specimen [17–19]. Tumor PD-L1 expression levels were determined by observing complete circumferential or partial linear expression (at any intensity) of PD-L1 on the cell membrane of viable tumor-cells using the laboratory-developed test with PD-L1 clone 22C3, stained on the Dako Autolink stainer. Assessment of expression levels was performed in sections that include at least 100 tumor cells that can be evaluated. The percentage of stained tumor cells in the entire specimen were scored on a continuous level.

Formalin-fixed paraffin-embedded (FFPE) resected tumor tissues and mediastinal lymph nodes (LN) were first assessed for the presence of tumor cells as part of routine care. Slides were stained with double immunohistochemistry for Ki67/CD8 and FOXP3/PD-1 using the BenchMark ULTRA system (Ventana), following the programmed double staining protocol and slides were covered using the Tissue-Tek Film® Coverslip.

Peripheral blood mononuclear cells (PBMC) were sampled at baseline, at the time of surgery, and at ca 12 weeks post-surgery. PBMC were isolated by density-gradient centrifugation with BD Vacutainer CPT tubes (BD Biosciences), and cryopreserved until further use as previously described [20]. Samples from baseline and time of surgery were thawed and surface stained with the following antibodies; CD3 [PerCP-Cy5.5], CD4 [AF700], CD8[V500], CD25 [APC], CD45RA [APC-H7], TIGIT [BV650],PD-1 [PE-Cy7], CD127 [BV421], PD-1 [BV786 (*BD Bioscience, USA*), HLA-DR [BV786], CD39 [PCF594], LAG3 [PE-Cy7](*eBioscience, USA)*. Clones utilized for these antibodies are shown in the supplementary data.

Additional intracellular staining was performed using the anti-human FoxP3 staining set (eBioscience, USA) with the following antibodies: Ki67 [FITC], CTLA-4 [PE-CF594 *(BD Bioscience, USA*)], and FoxP3 [PE] (*eBioscience, USA*) [PE]. Samples were acquired with an LSR Fortessa flow cytometer (BC Biosciences). Data was analyzed with Flowjo software.

# Supplementary figures

## Gating strategy PBMC subsets

| 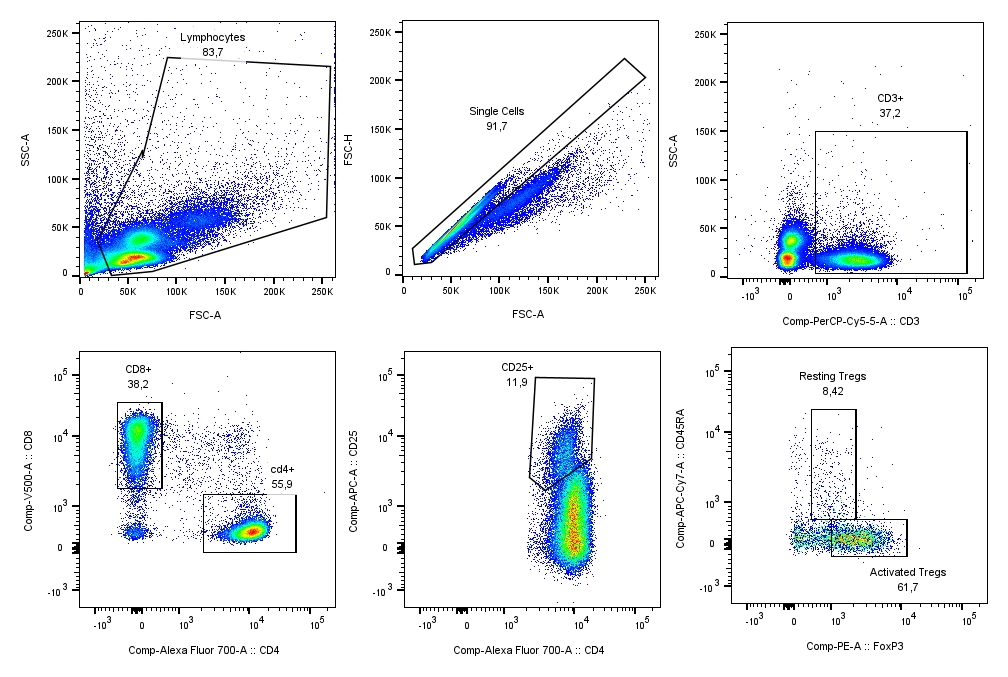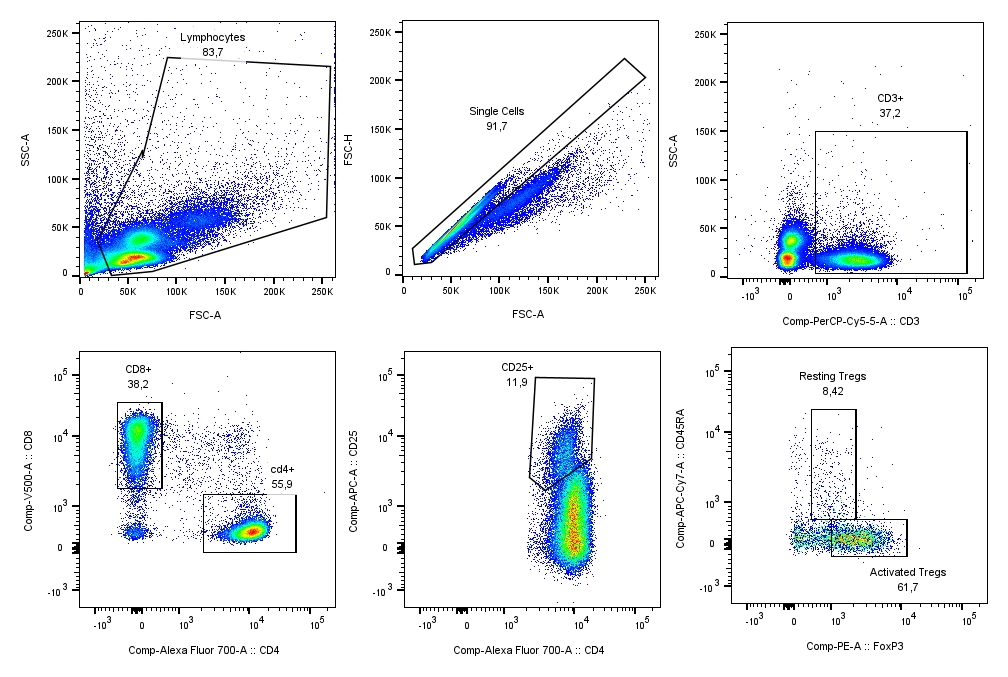 |
| --- |
| **Supplementary figure 1** (Online only): Gating strategy PBMC subsets. Blue arrows indicate the subsequent gating steps. Regulatory T cells (Treg) gating, as displayed in bottom right panel, is based on the publication by Miyara (Miyara et al, Immunity 30, 899–911 (2009)). Treg subset as presented in this manuscript are the Activated Treg and Resting Treg gates combined. |

## CD8 T cell immune checkpoint tests

| **A**   |
| --- |
| **B**   |
| **C**   |
| **Supplementary figure 2** (online only): Immune checkpoint expressions on CD8 T cells are shown. (A) The expression markers of LAG3, PD-1, TIGIT and CTLA4 (left pane = percentage of positive cells per overall CD8+ T cell population; right pane = mean fluorescence intensity) and the test p-values per checkpoint between patients with pCR (solid symbols) and without pCR (hollow symbols), at baseline, are shown. (B) For LAG3, TIGIT and CTLA4, the checkpoint expressions (median and range) at baseline, surgery, and 12 weeks post-surgery for patients without pCR (hollow symbols, N=5) and with pCR (solid symbols, N=6) are shown. P-values, testing differences between time points, are shown. (C) P-values, testing differences between tumor response groups per time point, are shown.  Utilized tests were 2way ANOVA with multiple comparisons, corrected using Šidák or Tukey, when appropriate.  **Abbreviations:** pCR = pathological complete response |

## Treg immune checkpoint tests

| **A**  **** |
| --- |
| **B**  **** |
| **C**  **** |
| **Supplementary figure 3 (**online only): Immune checkpoint expressions on Tregs are shown. (**A**) The expression markers of LAG3, PD-1, TIGIT and CTLA4 (left pane = percentage of positive cells per overall regulatory T cell population; right pane = mean fluorescence intensity) and the test p-values per checkpoint between patients with pCR (solid symbols) and without pCR (hollow symbols), at baseline, are shown. (**B**) For LAG3, TIGIT and CTLA4, the checkpoint expressions (median and range) at baseline, surgery, and 12 weeks post-surgery for patients without pCR (hollow symbols, N=5) and with pCR (solid symbols, N=6) are shown. P-values, testing differences between time points, are shown. (**C**) P-values, testing differences between tumor response groups per time point, are shown. Utilized tests were 2way ANOVA with multiple comparisons, corrected using Šidák or Tukey, when appropriate.  **Abbreviations:** pCR = pathological complete response |

## CD8+ T cell marker tests

| **A**  **** |
| --- |
| **B**  **** |
| **C**  **** |
| **Supplementary figure 4** (online only): Markers for proliferation (Ki67), T cell activation (HLADR) and tumor-association (CD39) on CD8 T cells are shown. (**A**) Floating bars (median and range) for the grouped CD8 T cells (pCR plus non-pCR patients) at baseline, surgery and 12 weeks post-surgery, and p-values, testing differences between these time points for each T cell marker, are shown. (**B**) Box and whisker plots (median and range) for patients with pCR (solid symbols, N=6) and without pCR (hollow symbols, N=5) are shown. P-values, testing differences between time points per tumor response group, are shown. (**C**) P-values, testing differences between tumor response groups per time point, are shown. Utilized tests were 1way and 2way ANOVA with multiple comparisons, corrected using Šidák or Tukey, when appropriate.  **Abbreviations:** pCR = pathological complete response |

## Treg marker tests

| **A**  **** |
| --- |
| **B**  **** |
| **C**  **** |
| **Supplementary figure 5** (online only): Markers for proliferation (Ki67), T cell activation (HLADR) and tumor-association (CD39) on regulatory cells are shown. (**A**) Floating bars (median and range) for the grouped Tregs (pCR plus non-pCR patients) at baseline, surgery and 12 weeks post-surgery, and p-values, testing differences between these time points for each T cell marker, are shown. (**B**) Box and whisker plots (median and range) for patients with pCR (hollow symbols, N=6) and without pCR (solid symbols, N=5) are shown. P-values, testing differences between time points per tumor response group, are shown. (**C**) P-values, testing differences between tumor response groups per time point, are shown. Utilized tests were 1way and 2way ANOVA with multiple comparisons, corrected using Šidák or Tukey, when appropriate.  **Abbreviations:** pCR = pathological complete response |

Survival plot

| **** |
| --- |
| **Supplementary figure 6** (online only): Kaplan-Meier plot of EFS (blue line) and OS (red line) of the intention to treat population is shown, as per August 2023.  **Abbreviations:** EFS = event-free survival; OS = overall survival |

# Supplementary tables

## Lung cancer molecular analysis panel

| Lung Cancer panel | |  |  |  |  |
| --- | --- | --- | --- | --- | --- |
| ABL1 | CTNNB1 | FGFR3 | KRAS | NRAS | ROS1 |
| AKT1 | EGFR | IDH1 | MAP2K1 | PIK3CA | STK11 |
| ALK | ERBB2 | IDH2 | MET | RB1 | TP53 |
| BRAF | FGFR1 | JAK2 | METex14skip | RET |  |
| CDKN2A | FGFR2 | KIT | NOTCH1 | RIT1 |  |
| Supplementary table 1. 28 genes, 150 amplicons, 15.09kb target, STK11 and TP53 are full gene coverages | | | | | |

## Clones utilized per each antibody

| Antibody | Company | Catalog number | Clone |
| --- | --- | --- | --- |
| CD3 PerCP-Cy5.5 | BD Biosciences | 332771 | SK7 |
| CD4 AF700 | BD Pharmingen | 557922 | RPA-T4 |
| CD8 V500 | BD Horizon | 561618 | SK1 |
| CD25 APC | BD | 340907 | 2A3 |
| CD45RA APC-H7 | BD Pharmingen | 560674 | HI100 |
| HLA-DR BV786 | Biolegend | 307642 | IL243 |
| TIGIT BV650 | BD OptiBuild | 747840 | 741182 |
| LAG3 PE-Cy7 | eBioscience | 25-2239-42 | 3SD223H |
| PD-1 BV786 | BD Horizon | 563789 | EH12.2 |
| CD39 PCF594 | Biolegend | 328224 | A1 |
| Ki67 FITC | BD Pharmingen | 556026 | B56 |
| CTLA-4 PE-CF594 | BD Horizon | 562742 | BNI3 |
| FoxP3 PE | eBioscience | 12-4776-42 | PCH101 |

| **Supplementary table 2.** Per antibody, the list of clone, company, and catalog number. (Online only) |
| --- |

## Baseline characteristics per pCR

| **Factor** | **Category** | **Total**  **n (%)** | **pCR**  **n (%)** | **No pCR**  **n (%)** | ***P*-value^#^** |
| --- | --- | --- | --- | --- | --- |
| Age (median, range) |  | 63 (43-73) | 63 (43-70) | 66 (51-73) | .169 |
| BMI (median, range) |  | 27 (19-33) | 27 (21-33) | 27 (19-29) | .851 |
| Sex | Male | 10 (40%) | 6 (40%) | 4 (40%) | 1.000 |
|  | Female | 15 (60%) | 9 (60%) | 6 (60%) |  |
| Smoking status* | Never | 1 (4%) | 0 (0%) | 1 (10%) | .262 |
|  | Former | 10 (40%) | 5 (33%) | 5 (50%) |  |
|  | Current | 14 (56%) | 10 (67%) | 4 (40%) |  |
| AID | Yes | 2 (8%) | 1 (7%) | 1 (10%) | .763 |
|  | No | 23 (92%) | 14 (93%) | 9 (90%) |  |
| ECOG | 0 | 15 (60%) | 8 (53%) | 7 (70%) | .405 |
|  | 1 | 10 (40%) | 7 (47%) | 3 (30%) |  |
| T-stage | T3 | 10 (40%) | 4 (27%) | 6 (60%) | .096 |
|  | T4 | 15 (60%) | 11 (73%) | 4 (40%) |  |
| N-stage | N0 | 15 (60%) | 10 (67%) | 5 (50%) | .707 |
|  | N1 | 8 (32%) | 4 (27%) | 4 (40%) |  |
|  | N2 | 2 (8%) | 1 (7%) | 1 (10%) |  |
| Histology | Non-squamous | 18 (72%) | 10 (67%) | 8 (80%) | .467 |
|  | Squamous | 7 (28%) | 5 (33%) | 2 (20%) |  |
| PD-L1 | 50% or above | 11 (44%) | 6 (40%) | 5 (50%) | .235 |
|  | 1-49% | 2 (8%) | 2 (13%) | 0 (0%) |  |
|  | less than 1% | 4 (16%) | 1 (7%) | 3 (30%) |  |
|  | unknown | 8 (32%) | 6 (40%) | 2 (20%) |  |
| AGA | Absent | 24 (96%) | 15 (100%) | 9 (90%) | .211 |
|  | Present | 1 (4%)**^$^** | 0 (0%) | 1 (10%) |  |
| Chemotherapy | 1 cycle | 1 (4%) | 0 (0%) | 1 (10%) | .337 |
|  | 2 cycles | 23 (92%) | 14 (93%) | 9 (90%) |  |
|  | 3 cycles | 1 (4%) | 1 (7%) | 0 (0%) |  |
| Immunotherapy | 1 cycle | 2 (8%) | 0 (0%) | 2 (20%) | .071 |
|  | 2 cycles | 23 (92%) | 15 (100%) | 8 (80%) |  |
| Radiotherapy | 60Gy | 2 (8%) | 1 (7%) | 1 (10%) | .763 |
|  | 50Gy | 23 (92%) | 14 (93%) | 9 (90%) |  |
|  | less than 50Gy | 0 (0%) | 0 (0%) | 0 (0%) |  |

| **Supplementary table 3.** Baseline characteristics of the operated patient population grouped per pCR outcome is shown. (Online only)  **Comments**: (*) Never <100 cigarettes in life-time, Former = stopped smoking for >1 year, Current = active smoker or has stopped for <1 year. (**#**) The 2-sided *P*-value is based on a student t-test for scale variables and on a χ^2^ test for categorical variables. (**$**) In one patient, an actionable EGFR mutation was found only on the post-resection tumor specimen, this was not identified on the baseline biopsy due to low yield.  **Abbreviations**: pCR = pathological complete response, BMI = body mass index, AID = autoimmune disease in past medical history, ECOG = performance status scale according to the Eastern Cooperative Oncology Group, PD-L1 = programmed death ligand-1, AGA = actionable genomic alteration. |
| --- |

## Treatment-emergent adverse events

| **TABLE of TEAEs** | **Any grade**  **n (%)** | **Grade 1-2**  **n** | **Grade 3-4**  **n** | **Grade 5**  **n** |
| --- | --- | --- | --- | --- |
| Any | 30 (100%) | 30 | 22 | 3 |
| Dermatitis/rash | 25 (83%) | 23 | 2 | 0 |
| Apetite loss/Nausea/Vomiting | 25 (83%) | 23 | 2 | 0 |
| Pain | 24 (80%) | 23 | 1 | 0 |
| Anemia | 23 (77%) | 21 | 2 | 0 |
| Fatigue | 20 (67%) | 18 | 2 | 0 |
| Constipation/diarrhea | 18 (60%) | 18 | 0 | 0 |
| Transaminitis/Hepatitis | 17 (57%) | 13 | 4 | 0 |
| Leucopenia | 17 (57%) | 10 | 7 | 0 |
| Electrolyte disorders | 17 (57%) | 16 | 1 | 0 |
| Esophagitis | 16 (53%) | 15 | 1 | 0 |
| Cough | 15 (50%) | 15 | 0 | 0 |
| Thrombocytopenia | 14 (47%) | 9 | 5 | 0 |
| Nervous disorders | 12 (40%) | 12 | 0 | 0 |
| Dyspnea | 12 (40%) | 8 | 4 | 0 |
| Alk Phos increased | 11 (37%) | 11 | 0 | 0 |
| Thyroid disorders | 10 (33%) | 10 | 0 | 0 |
| Fever | 9 (30%) | 9 | 0 | 0 |
| GGT increased | 8 (27%) | 6 | 2 | 0 |
| CRP increased | 7 (23%) | 7 | 0 | 0 |
| Urinary tract infection | 6 (20%) | 6 | 0 | 0 |
| LDH increased | 6 (20%) | 6 | 0 | 0 |
| Pneumonitis | 5 (17%) | 2 | 2 | 1 |
| Lymphopenia | 5 (17%) | 4 | 1 | 0 |
| Insomnia | 4 (13%) | 4 | 0 | 0 |
| Heart rhythm disorders | 4 (13%) | 4 | 0 | 0 |
| Dry skin | 4 (13%) | 4 | 0 | 0 |
| Infusion related reaction | 3 (10%) | 3 | 0 | 0 |
| Anxiety/confusion | 3 (10%) | 1 | 2 | 0 |
| Wound dehiscense | 2 (7%) | 2 | 0 | 0 |
| Subcutaneous emphysema | 2 (7%) | 2 | 0 | 0 |
| Respiratory tract infection | 2 (7%) | 2 | 0 | 0 |
| Renal function decreased | 2 (7%) | 2 | 0 | 0 |
| Pericarditis | 2 (7%) | 2 | 0 | 0 |
| Neutropenia | 2 (7%) | 1 | 1 | 0 |
| Hoarseness | 2 (7%) | 2 | 0 | 0 |
| Dry mouth | 2 (7%) | 2 | 0 | 0 |
| Chylothorax | 2 (7%) | 2 | 0 | 0 |
| Blurred vision | 2 (7%) | 2 | 0 | 0 |
| Allergic reaction | 2 (7%) | 2 | 0 | 0 |
| Vaginal infection | 1 (3%) | 1 | 0 | 0 |
| Thromboembolic event | 1 (3%) | 1 | 0 | 0 |
| Thrombocytosis | 1 (3%) | 1 | 0 | 0 |
| Pneumothorax | 1 (3%) | 1 | 0 | 0 |
| Pleural infection | 1 (3%) | 1 | 0 | 0 |
| Pancreatitis | 1 (3%) | 0 | 1 | 0 |
| Myositis | 1 (3%) | 0 | 1 | 0 |
| Mucositis | 1 (3%) | 1 | 0 | 0 |
| Melaena | 1 (3%) | 1 | 0 | 0 |
| Lipase/amylase increased | 1 (3%) | 1 | 0 | 0 |
| Leucocytosis | 1 (3%) | 1 | 0 | 0 |
| Keratitis | 1 (3%) | 1 | 0 | 0 |
| Hypoxia | 1 (3%) | 0 | 0 | 1 |
| Hypotension | 1 (3%) | 1 | 0 | 0 |
| Hypomagnesemia | 1 (3%) | 1 | 0 | 0 |
| Hyperhidrosis | 1 (3%) | 1 | 0 | 0 |
| Heart failure/valve disorders | 1 (3%) | 0 | 1 | 0 |
| Fracture | 1 (3%) | 1 | 0 | 0 |
| Fall | 1 (3%) | 1 | 0 | 0 |
| Edema limbs | 1 (3%) | 1 | 0 | 0 |
| Diplopia | 1 (3%) | 1 | 0 | 0 |
| Bloating | 1 (3%) | 1 | 0 | 0 |
| Bacteremia | 1 (3%) | 1 | 0 | 0 |
| Atelectasis | 1 (3%) | 1 | 0 | 0 |
| Anosmia | 1 (3%) | 1 | 0 | 0 |
| Alopecia | 1 (3%) | 1 | 0 | 0 |

| **Supplementary table 4.** Treatment-emergent adverse events. (Online only)  TEAEs monitored in all 29 patients from the intention-to-treat population, occurring in the time from start of therapy until 90 days post-surgery or if no surgery was performed until 180 days after start of therapy, are shown.  **Abbreviations**: TEAE = treatment-emergent adverse events |
| --- |

## Treatment-related adverse events

| **TABLE of TRAEs** | **Any grade**  **n (%)** | **Grade 1-2**  **n** | **Grade 3-4**  **n** | **Grade 5**  **n** |
| --- | --- | --- | --- | --- |
| Any | 30 (100%) | 30 | 21 | 1 |
| Dermatitis/rash | 24 (80%) | 22 | 2 | 0 |
| Apetite loss/Nausea/Vomiting | 21 (70%) | 19 | 2 | 0 |
| Anemia | 21 (70%) | 19 | 2 | 0 |
| Fatigue | 18 (60%) | 17 | 1 | 0 |
| Transaminitis/Hepatitis | 17 (57%) | 13 | 4 | 0 |
| Leucopenia | 17 (57%) | 10 | 7 | 0 |
| Esophagitis | 16 (53%) | 15 | 1 | 0 |
| Electrolyte disorders | 16 (53%) | 15 | 1 | 0 |
| Constipation/diarrhea | 16 (53%) | 16 | 0 | 0 |
| Thrombocytopenia | 14 (47%) | 9 | 5 | 0 |
| Pain | 13 (43%) | 13 | 0 | 0 |
| Alk Phos increased | 11 (37%) | 11 | 0 | 0 |
| Thyroid disorders | 10 (33%) | 10 | 0 | 0 |
| Dyspnea | 10 (33%) | 7 | 3 | 0 |
| Cough | 10 (33%) | 10 | 0 | 0 |
| GGT increased | 8 (27%) | 6 | 2 | 0 |
| Fever | 6 (20%) | 6 | 0 | 0 |
| Nervous disorders | 5 (17%) | 5 | 0 | 0 |
| Lymphopenia | 5 (17%) | 4 | 1 | 0 |
| LDH increased | 5 (17%) | 5 | 0 | 0 |
| Pneumonitis | 4 (13%) | 2 | 1 | 1 |
| Dry skin | 4 (13%) | 4 | 0 | 0 |
| Insomnia | 3 (10%) | 3 | 0 | 0 |
| Infusion related reaction | 3 (10%) | 3 | 0 | 0 |
| CRP increased | 3 (10%) | 3 | 0 | 0 |
| Wound dehiscense | 2 (7%) | 2 | 0 | 0 |
| Subcutaneous emphysema | 2 (7%) | 2 | 0 | 0 |
| Neutropenia | 2 (7%) | 1 | 1 | 0 |
| Dry mouth | 2 (7%) | 2 | 0 | 0 |
| Blurred vision | 2 (7%) | 2 | 0 | 0 |
| Allergic reaction | 2 (7%) | 2 | 0 | 0 |
| Vaginal infection | 1 (3%) | 1 | 0 | 0 |
| Urinary tract infection | 1 (3%) | 1 | 0 | 0 |
| Thrombocytosis | 1 (3%) | 1 | 0 | 0 |
| Renal function decreased | 1 (3%) | 1 | 0 | 0 |
| Pneumothorax | 1 (3%) | 1 | 0 | 0 |
| Pleural infection | 1 (3%) | 1 | 0 | 0 |
| Pericarditis | 1 (3%) | 1 | 0 | 0 |
| Pancreatitis | 1 (3%) | 0 | 1 | 0 |
| Myositis | 1 (3%) | 0 | 1 | 0 |
| Mucositis | 1 (3%) | 1 | 0 | 0 |
| Lipase/amylase increased | 1 (3%) | 1 | 0 | 0 |
| Leucocytosis | 1 (3%) | 1 | 0 | 0 |
| Hypotension | 1 (3%) | 1 | 0 | 0 |
| Hypomagnesemia | 1 (3%) | 1 | 0 | 0 |
| Hypomagnemesia | 1 (3%) | 1 | 0 | 0 |
| Hoarseness | 1 (3%) | 1 | 0 | 0 |
| Heart failure/valve disorders | 1 (3%) | 0 | 1 | 0 |
| Edema limbs | 1 (3%) | 1 | 0 | 0 |
| Bloating | 1 (3%) | 1 | 0 | 0 |
| Anxiety/confusion | 1 (3%) | 0 | 1 | 0 |
| Anosmia | 1 (3%) | 1 | 0 | 0 |
| Alopecia | 1 (3%) | 1 | 0 | 0 |

| **Supplementary table 5**. Treatment-related adverse events. (Online only)  TRAEs monitored in all 30 patients that started induction therapy, occurring in the time from start of therapy until 90 days post-surgery or if no surgery was performed until 180 days after start of therapy, are shown.  **Abbreviations**: TRAE = treatment-related adverse events |
| --- |

## Pathological response

| **Patient nr** | **PD-L1** | **Tumor regression** |
| --- | --- | --- |
| 15 | N/A | N/A |
| 19 | N/A | N/A |
| 26 | 5% | N/A |
| 32 | N/A | N/A |
| 22 | 0% | -30% |
| 21 | N/A | -40% |
| 31 | 70% | -60% |
| 03 | N/A | -70% |
| 27 | N/A | -70% |
| 09 | 0% | -85% |
| 02 | 100% | -90% |
| 07 | 100% | -99% |
| 16 | 0% | -99% |
| 18 | N/A | -99% |
| 29 | N/A | -100% |
| 05 | 20% | -100% |
| 24 | N/A | -100% |
| 20 | N/A | -100% |
| 01 | 0% | -100% |
| 17 | N/A | -100% |
| 23 | 100% | -100% |
| 08 | 90% | -100% |
| 12 | 15% | -100% |
| 28 | N/A | -100% |
| 25 | N/A | -100% |
| 14 | N/A | -100% |
| 06 | 90% | -100% |
| 11 | N/A | -100% |
| 10 | N/A | -100% |
| **Supplementary table 6**. Pathological tumor response and tumor PD-L1 at baseline. (Online only) | | |

## Mutation analysis

| **Patient nr** | **Detailed molecular results (if present)** |
| --- | --- |
| 1 | TP53 exon 7 (c.730G>T; p.(Gly244Cys); VAF 83%) |
| 2 | SQUAMOUS |
| 3 | EGFR exon 21 (c.2573T>G; p.(Leu858Arg); VAF 48%);  TP53 exon 5 (c.437_440del; p.(Trp146Leufs*23); VAF 63%) |
| 4 | SCREEN FAILURE |
| 5 | TP53 exon 5 (c.463A>C, p.T155P, in 41% of reads);  CDKN2A exon 2 (c.332delG, p.G111Afs*35, in 60% of reads) |
| 6 | SQUAMOUS |
| 7 | TP53 exon 7 (c.747G>T; p.(Arg249Ser); 56%VAF) |
| 8 | TP53 exon 7 (c.733G>T, p.G245C, in 51% of reads) |
| 9 | STK11 exon 4 (c.523A>T; p.(Lys175Ter); VAF 45%),  TP53 exon 8 (c.814G>C; p.(Val272Leu); VAF 45%) |
| 10 | SQUAMOUS |
| 11 | SQUAMOUS |
| 12 | TP53 exon 6 (c.609_610delinsAT; p.(Glu204Ter); VAF 14%);  TP53 exon 8 (c.854A>T; p.(Glu285Val); VAF 11%) |
| 13 | SCREEN FAILURE |
| 14 | SQUAMOUS |
| 15 | CTNNB1 exon 3 (c.98C>T; p.(Ser33Phe); VAF 56%),  STK11 exon 1 (c.125del; p.(Ala43Profs*8); VAF 40%) |
| 16 | STK11 exon 8 (c.923G>A; p. (Trp308Ter); VAF 81%),  TP53 exon 7 (c.742C>T; p. (Arg248Trp); VAF 79%) |
| 17 | insufficient tumor at baseline and pCR postresection |
| 18 | SQUAMOUS |
| 19 | NRAS exon 2 (c.38G>A, p.G13D), TP53 exon 10 (c.1010G>T, p.R337L) |
| 20 | TP53 exon 4 (c.375G>T; p.?); splice site mutatie; VAF 43%) |
| 21 | CDKN2A exon 2 (c.347A>G; p.(Asp116Gly); VAF 24%),  STK11 exon 4 (c.488G>A; p.(Gly163Asp); VAF 24%),  TP53 exon 8 (c.814G>T; p.(Val272Leu); VAF 22%) |
| 22 | ERBB2 exon 20 (c.2331_2339dup, p.G778_P780dup) in 31% of reads |
| 23 | TP53 exon 6 (c.607G>T; p.(Val203Leu); VAF18%) |
| 24 | insufficient tumor at baseline and pCR postresection |
| 25 | SQUAMOUS |
| 26 | TP53 exon 6 (c.638G>T; p.(Arg213Leu); VAF 42%) |
| 27 | TP53 exon 8 (c.808T>C, p.F270L) in 21% of reads |
| 28 | KRAS exon 2 (c.35G>A; p.(Gly12Asp); VAF 61%),  PIK3CA exon 10 (c.1633G>A; p.(Glu545Lys); VAF 17%) |
| 29 | no mutations found |
| 30 | BRAF exon 15 (c.1799T>A; p.(Val600Glu); VAF 8,4%),  PIK3CA exon 21 (c.3145G>C; p.(Gly1049Arg); VAF 7,8%),  TERT promoter (c.-124C>T; ook wel C228T; VAF 21%),  MAP2K4 exon 1 (c.23G>T; p.(Gly8Val); VAF 6,5%) |
| 31 | NRAS exon 3 (c.182A>T; p.(Gln61Leu); VAF 34%),  TP53 exon 5 (c.469G>T; p.(Val157Phe); VAF 42%) |
| 32 | SQUAMOUS |
| **Supplementary table 7.** Results of the molecular analysis of the thoracic tumors (Online only) | |

## Survival outcomes

| **Patient nr** | **Event** | **Recurrence** | **EFS  (days)** | **DFS**  **(days)** | **OS**  **(days)** |
| --- | --- | --- | --- | --- | --- |
| 01 | No recurrence observed | No | 1287 | 1210 | 1287 |
| 02 | No recurrence observed | No | 1042 | 943 | 1042 |
| 03 | Recurrence with only brain metastases | Yes | 342 | 253 | 346 |
| 05 | Recurrence with only brain metastases | Yes | 284 | 194 | 464 |
| 06 | No recurrence observed | No | 1150 | 1058 | 1150 |
| 07 | Recurrence with only brain metastases | Yes | 155 | 70 | 244 |
| 08 | Bilateral intrapulmonary nodules, not pathologically confirmed, considered as intrapulmonary metastases, and received systemic therapy | Yes | 196 | 113 | 1074 |
| 09 | No recurrence observed | No | 1058 | 971 | 810 |
| 10 | No recurrence observed | No | 1045 | 968 | 1045 |
| 11 | No recurrence observed | No | 1075 | 995 | 1075 |
| 12 | Recurrence with adrenal metastasis and abdominal lymph nodes | Yes | 657 | 565 | 1071 |
| 14 | No recurrence observed | No | 960 | 879 | 960 |
| 15 | Recurrence with pleural metastases after induction therapy, did not undergo surgery | Yes | 56 | N/A | 239 |
| 16 | No recurrence observed | No | 823 | 739 | 823 |
| 17 | No recurrence observed | No | 924 | 841 | 924 |
| 18 | No recurrence observed | No | 800 | 723 | 800 |
| 19 | Died due to COVID-19 during induction therapy | No | 29 | N/A | 29 |
| 20 | Recurrence with only brain metastases | Yes | 688 | 611 | 905 |
| 21 | No recurrence observed | No | 932 | 821 | 901 |
| 22 | No recurrence observed | No | 770 | 692 | 770 |
| 23 | No recurrence observed | No | 799 | 716 | 799 |
| 24 | No recurrence observed | No | 356 | 272 | 356 |
| 25 | No recurrence observed | No | 721 | 643 | 721 |
| 26 | Did not receive an operation per MDT recommendation, received durvalumab, no recurrence observed to date. | No | 711 | N/A | 713 |
| 27 | No recurrence observed | No | 669 | 589 | 676 |
| 28 | No recurrence observed | No | 665 | 580 | 672 |
| 29 | Died due to pneumonitis, no recurrence observed. | No | 174 | 96 | 174 |
| 30 | Recurrence with only brain metastases | Yes | 101 | 2 | 114 |
| 31 | No recurrence observed | No | 321 | 230 | 321 |
| 32 | Developed intrapulmonary nodules, therefore patient was not operated, ultimately the nodules regressed spontaneously | No | 203 | N/A | 203 |
| **Supplementary table 8.** Survival outcomes for the intention-to-treat population. Events and recurrence state is shown as per August 2023.  **Abbreviations**: EFS: event free survival, defined as time from study inclusion to growth of tumor or death; DFS: disease free survival, defined as the time from resection to recurrence of tumor or death; OS: overall survival, defined as the time from study inclusion to death; N/A: not applicable. (Online only) | | | | | |

# PBMC analysis - test comparison tables

### CD8+ T cells at baseline, test differences between immune checkpoints (Fig 4A)

| Number of families | 1 |  |  |  |  |  |
| --- | --- | --- | --- | --- | --- | --- |
| Number of comparisons per family | 6 |  |  |  |  |  |
| Alpha | 0,05 |  |  |  |  |  |
|  |  |  |  |  |  |  |
| Tukey's multiple comparisons test | Mean Diff, | 95,00% CI of diff, | Below threshold? | Summary | Adjusted P Value |  |
| LAG3 vs. PD-1 | -29,92 | -39,03 to -20,81 | Yes | **** | <0,0001 | A-B |
| LAG3 vs. TIGIT | -28,80 | -37,91 to -19,68 | Yes | **** | <0,0001 | A-C |
| LAG3 vs. CTLA4 | 0,3723 | -8,742 to 9,487 | No | ns | 0,9995 | A-D |
| PD-1 vs. TIGIT | 1,123 | -7,991 to 10,24 | No | ns | 0,9877 | B-C |
| PD-1 vs. CTLA4 | 30,29 | 21,18 to 39,41 | Yes | **** | <0,0001 | B-D |
| TIGIT vs. CTLA4 | 29,17 | 20,05 to 38,28 | Yes | **** | <0,0001 | C-D |

| Test details | Mean 1 | Mean 2 | Mean Diff, | SE of diff, | n1 | n2 | q | DF |
| --- | --- | --- | --- | --- | --- | --- | --- | --- |
| LAG3 vs. PD-1 | 4,388 | 34,31 | -29,92 | 3,425 | 13 | 13 | 12,36 | 48 |
| LAG3 vs. TIGIT | 4,388 | 33,18 | -28,80 | 3,425 | 13 | 13 | 11,89 | 48 |
| LAG3 vs. CTLA4 | 4,388 | 4,015 | 0,3723 | 3,425 | 13 | 13 | 0,1537 | 48 |
| PD-1 vs. TIGIT | 34,31 | 33,18 | 1,123 | 3,425 | 13 | 13 | 0,4638 | 48 |
| PD-1 vs. CTLA4 | 34,31 | 4,015 | 30,29 | 3,425 | 13 | 13 | 12,51 | 48 |
| TIGIT vs. CTLA4 | 33,18 | 4,015 | 29,17 | 3,425 | 13 | 13 | 12,05 | 48 |

### Tregs at baseline, test differences between immune checkpoints (Fig 4A)

| Number of families | 1 |  |  |  |  |  |
| --- | --- | --- | --- | --- | --- | --- |
| Number of comparisons per family | 6 |  |  |  |  |  |
| Alpha | 0,05 |  |  |  |  |  |
|  |  |  |  |  |  |  |
| Tukey's multiple comparisons test | Mean Diff, | 95,00% CI of diff, | Below threshold? | Summary | Adjusted P Value |  |
| LAG3 vs. PD-1 | -31,72 | -41,62 to -21,83 | Yes | **** | <0,0001 | A-B |
| LAG3 vs. TIGIT | -58,68 | -68,58 to -48,78 | Yes | **** | <0,0001 | A-C |
| LAG3 vs. CTLA4 | -78,49 | -88,39 to -68,59 | Yes | **** | <0,0001 | A-D |
| PD-1 vs. TIGIT | -26,95 | -36,85 to -17,05 | Yes | **** | <0,0001 | B-C |
| PD-1 vs. CTLA4 | -46,76 | -56,66 to -36,86 | Yes | **** | <0,0001 | B-D |
| TIGIT vs. CTLA4 | -19,81 | -29,71 to -9,908 | Yes | **** | <0,0001 | C-D |

| Test details | Mean 1 | Mean 2 | Mean Diff, | SE of diff, | n1 | n2 | q | DF |
| --- | --- | --- | --- | --- | --- | --- | --- | --- |
| LAG3 vs. PD-1 | 2,022 | 33,75 | -31,72 | 3,720 | 13 | 13 | 12,06 | 48 |
| LAG3 vs. TIGIT | 2,022 | 60,70 | -58,68 | 3,720 | 13 | 13 | 22,31 | 48 |
| LAG3 vs. CTLA4 | 2,022 | 80,51 | -78,49 | 3,720 | 13 | 13 | 29,84 | 48 |
| PD-1 vs. TIGIT | 33,75 | 60,70 | -26,95 | 3,720 | 13 | 13 | 10,25 | 48 |
| PD-1 vs. CTLA4 | 33,75 | 80,51 | -46,76 | 3,720 | 13 | 13 | 17,78 | 48 |
| TIGIT vs. CTLA4 | 60,70 | 80,51 | -19,81 | 3,720 | 13 | 13 | 7,531 | 48 |

### CD8+ T cells at 3 timepoints, test differences between immune checkpoints (Fig 4B)

#### CD8+ T cells - LAG3

##### Timepoint comparison

| Tukey's multiple comparisons test | Mean Diff, | 95,00% CI of diff, | Below threshold? | Summary | Adjusted P Value |  |  |  |
| --- | --- | --- | --- | --- | --- | --- | --- | --- |
|  |  |  |  |  |  |  |  |  |
| Baseline vs. Surgery | 0,4418 | -1,444 to 2,328 | No | ns | 0,8008 |  |  |  |
| Baseline vs. Post-surgery | -1,165 | -4,224 to 1,893 | No | ns | 0,5674 |  |  |  |
| Surgery vs. Post-surgery | -1,607 | -4,545 to 1,330 | No | ns | 0,3317 |  |  |  |
|  |  |  |  |  |  |  |  |  |
|  |  |  |  |  |  |  |  |  |
| Test details | Mean 1 | Mean 2 | Mean Diff, | SE of diff, | N1 | N2 | q | DF |
|  |  |  |  |  |  |  |  |  |
| Baseline vs. Surgery | 4,671 | 4,229 | 0,4418 | 0,6879 | 11 | 11 | 0,9083 | 10,00 |
| Baseline vs. Post-surgery | 4,671 | 5,836 | -1,165 | 1,116 | 11 | 11 | 1,477 | 10,00 |
| Surgery vs. Post-surgery | 4,229 | 5,836 | -1,607 | 1,072 | 11 | 11 | 2,121 | 10,00 |

| Tukey's multiple comparisons test | Mean Diff, | 95,00% CI of diff, | Below threshold? | Summary | Adjusted P Value |  |  |  |
| --- | --- | --- | --- | --- | --- | --- | --- | --- |
|  |  |  |  |  |  |  |  |  |
| Non pCR |  |  |  |  |  |  |  |  |
| Baseline vs. Surgery | 2,034 | 0,2007 to 3,867 | Yes | * | 0,0360 |  |  |  |
| Baseline vs. Post-surgery | -1,122 | -7,905 to 5,661 | No | ns | 0,8329 |  |  |  |
| Surgery vs. Post-surgery | -3,156 | -9,964 to 3,652 | No | ns | 0,3270 |  |  |  |
|  |  |  |  |  |  |  |  |  |
| pCR |  |  |  |  |  |  |  |  |
| Baseline vs. Surgery | -0,8850 | -3,766 to 1,996 | No | ns | 0,6086 |  |  |  |
| Baseline vs. Post-surgery | -1,202 | -5,972 to 3,568 | No | ns | 0,7083 |  |  |  |
| Surgery vs. Post-surgery | -0,3167 | -3,642 to 3,009 | No | ns | 0,9490 |  |  |  |
|  |  |  |  |  |  |  |  |  |
|  |  |  |  |  |  |  |  |  |
| Test details | Mean 1 | Mean 2 | Mean Diff, | SE of diff, | N1 | N2 | q | DF |
|  |  |  |  |  |  |  |  |  |
| Non pCR |  |  |  |  |  |  |  |  |
| Baseline vs. Surgery | 6,122 | 4,088 | 2,034 | 0,5144 | 5 | 5 | 5,592 | 4,000 |
| Baseline vs. Post-surgery | 6,122 | 7,244 | -1,122 | 1,903 | 5 | 5 | 0,8337 | 4,000 |
| Surgery vs. Post-surgery | 4,088 | 7,244 | -3,156 | 1,910 | 5 | 5 | 2,336 | 4,000 |
|  |  |  |  |  |  |  |  |  |
| pCR |  |  |  |  |  |  |  |  |
| Baseline vs. Surgery | 3,462 | 4,347 | -0,8850 | 0,8855 | 6 | 6 | 1,413 | 5,000 |
| Baseline vs. Post-surgery | 3,462 | 4,663 | -1,202 | 1,466 | 6 | 6 | 1,159 | 5,000 |
| Surgery vs. Post-surgery | 4,347 | 4,663 | -0,3167 | 1,022 | 6 | 6 | 0,4382 | 5,000 |

##### pCR vs non-pCR comparison

| Šídák's multiple comparisons test | Mean Diff, | 95,00% CI of diff, | Below threshold? | Summary | Adjusted P Value |  |  |  |
| --- | --- | --- | --- | --- | --- | --- | --- | --- |
|  |  |  |  |  |  |  |  |  |
| Non pCR - pCR |  |  |  |  |  |  |  |  |
| Baseline | 2,660 | -2,295 to 7,616 | No | ns | 0,3347 |  |  |  |
| Surgery | -0,2587 | -3,824 to 3,307 | No | ns | 0,9956 |  |  |  |
| Post-surgery | 2,581 | -5,118 to 10,28 | No | ns | 0,7181 |  |  |  |
|  |  |  |  |  |  |  |  |  |
|  |  |  |  |  |  |  |  |  |
| Test details | Mean 1 | Mean 2 | Mean Diff, | SE of diff, | N1 | N2 | t | DF |
|  |  |  |  |  |  |  |  |  |
| Non pCR - pCR |  |  |  |  |  |  |  |  |
| Baseline | 6,122 | 3,462 | 2,660 | 1,493 | 5 | 6 | 1,782 | 5,776 |
| Surgery | 4,088 | 4,347 | -0,2587 | 1,215 | 5 | 6 | 0,2129 | 8,838 |
| Post-surgery | 7,244 | 4,663 | 2,581 | 2,570 | 5 | 6 | 1,004 | 8,094 |

#### CD8+ T cells - TIGIT

##### Timepoint comparison

| Tukey's multiple comparisons test | Mean Diff, | 95,00% CI of diff, | Below threshold? | Summary | Adjusted P Value |  |  |  |
| --- | --- | --- | --- | --- | --- | --- | --- | --- |
|  |  |  |  |  |  |  |  |  |
| Baseline vs. Surgery | -7,373 | -19,41 to 4,663 | No | ns | 0,2599 |  |  |  |
| Baseline vs. Post-surgery | -8,991 | -21,15 to 3,170 | No | ns | 0,1561 |  |  |  |
| Surgery vs. Post-surgery | -1,618 | -11,14 to 7,904 | No | ns | 0,8885 |  |  |  |
|  |  |  |  |  |  |  |  |  |
|  |  |  |  |  |  |  |  |  |
| Test details | Mean 1 | Mean 2 | Mean Diff, | SE of diff, | N1 | N2 | q | DF |
|  |  |  |  |  |  |  |  |  |
| Baseline vs. Surgery | 34,76 | 42,14 | -7,373 | 4,391 | 11 | 11 | 2,375 | 10,00 |
| Baseline vs. Post-surgery | 34,76 | 43,75 | -8,991 | 4,436 | 11 | 11 | 2,866 | 10,00 |
| Surgery vs. Post-surgery | 42,14 | 43,75 | -1,618 | 3,474 | 11 | 11 | 0,6588 | 10,00 |

| Tukey's multiple comparisons test | Mean Diff, | 95,00% CI of diff, | Below threshold? | Summary | Adjusted P Value |  |  |  |
| --- | --- | --- | --- | --- | --- | --- | --- | --- |
|  |  |  |  |  |  |  |  |  |
| Non pCR |  |  |  |  |  |  |  |  |
| Baseline vs. Surgery | -3,100 | -26,74 to 20,54 | No | ns | 0,8897 |  |  |  |
| Baseline vs. Post-surgery | -6,400 | -33,42 to 20,62 | No | ns | 0,6989 |  |  |  |
| Surgery vs. Post-surgery | -3,300 | -28,71 to 22,11 | No | ns | 0,8917 |  |  |  |
|  |  |  |  |  |  |  |  |  |
| pCR |  |  |  |  |  |  |  |  |
| Baseline vs. Surgery | -10,93 | -30,40 to 8,531 | No | ns | 0,2530 |  |  |  |
| Baseline vs. Post-surgery | -11,15 | -29,50 to 7,199 | No | ns | 0,2128 |  |  |  |
| Surgery vs. Post-surgery | -0,2167 | -10,23 to 9,800 | No | ns | 0,9973 |  |  |  |
|  |  |  |  |  |  |  |  |  |
|  |  |  |  |  |  |  |  |  |
| Test details | Mean 1 | Mean 2 | Mean Diff, | SE of diff, | N1 | N2 | q | DF |
|  |  |  |  |  |  |  |  |  |
| Non pCR |  |  |  |  |  |  |  |  |
| Baseline vs. Surgery | 40,98 | 44,08 | -3,100 | 6,632 | 5 | 5 | 0,6611 | 4,000 |
| Baseline vs. Post-surgery | 40,98 | 47,38 | -6,400 | 7,580 | 5 | 5 | 1,194 | 4,000 |
| Surgery vs. Post-surgery | 44,08 | 47,38 | -3,300 | 7,131 | 5 | 5 | 0,6545 | 4,000 |
|  |  |  |  |  |  |  |  |  |
| pCR |  |  |  |  |  |  |  |  |
| Baseline vs. Surgery | 29,58 | 40,52 | -10,93 | 5,982 | 6 | 6 | 2,585 | 5,000 |
| Baseline vs. Post-surgery | 29,58 | 40,73 | -11,15 | 5,639 | 6 | 6 | 2,796 | 5,000 |
| Surgery vs. Post-surgery | 40,52 | 40,73 | -0,2167 | 3,078 | 6 | 6 | 0,09954 | 5,000 |

##### pCR vs non-pCR comparison

| Šídák's multiple comparisons test | Mean Diff, | 95,00% CI of diff, | Below threshold? | Summary | Adjusted P Value |  |  |  |
| --- | --- | --- | --- | --- | --- | --- | --- | --- |
|  |  |  |  |  |  |  |  |  |
| Non pCR - pCR |  |  |  |  |  |  |  |  |
| Baseline | 11,40 | -8,751 to 31,54 | No | ns | 0,2781 |  |  |  |
| Surgery | 3,563 | -26,19 to 33,32 | No | ns | 0,9812 |  |  |  |
| Post-surgery | 6,647 | -18,93 to 32,23 | No | ns | 0,8479 |  |  |  |
|  |  |  |  |  |  |  |  |  |
|  |  |  |  |  |  |  |  |  |
| Test details | Mean 1 | Mean 2 | Mean Diff, | SE of diff, | N1 | N2 | t | DF |
|  |  |  |  |  |  |  |  |  |
| Non pCR - pCR |  |  |  |  |  |  |  |  |
| Baseline | 40,98 | 29,58 | 11,40 | 5,713 | 5 | 6 | 1,995 | 4,973 |
| Surgery | 44,08 | 40,52 | 3,563 | 10,17 | 5 | 6 | 0,3504 | 8,961 |
| Post-surgery | 47,38 | 40,73 | 6,647 | 8,732 | 5 | 6 | 0,7612 | 8,911 |

#### CD8+ T cells - CTLA4

##### Timepoint comparison

| Tukey's multiple comparisons test | Mean Diff, | 95,00% CI of diff, | Below threshold? | Summary | Adjusted P Value |  |  |  |
| --- | --- | --- | --- | --- | --- | --- | --- | --- |
|  |  |  |  |  |  |  |  |  |
| Baseline vs. Surgery | -1,930 | -8,222 to 4,362 | No | ns | 0,6875 |  |  |  |
| Baseline vs. Post-surgery | -0,4064 | -1,611 to 0,7979 | No | ns | 0,6377 |  |  |  |
| Surgery vs. Post-surgery | 1,524 | -3,731 to 6,778 | No | ns | 0,7144 |  |  |  |
|  |  |  |  |  |  |  |  |  |
|  |  |  |  |  |  |  |  |  |
| Test details | Mean 1 | Mean 2 | Mean Diff, | SE of diff, | N1 | N2 | q | DF |
|  |  |  |  |  |  |  |  |  |
| Baseline vs. Surgery | 4,040 | 5,970 | -1,930 | 2,295 | 11 | 11 | 1,189 | 10,00 |
| Baseline vs. Post-surgery | 4,040 | 4,446 | -0,4064 | 0,4393 | 11 | 11 | 1,308 | 10,00 |
| Surgery vs. Post-surgery | 5,970 | 4,446 | 1,524 | 1,917 | 11 | 11 | 1,124 | 10,00 |

| Tukey's multiple comparisons test | Mean Diff, | 95,00% CI of diff, | Below threshold? | Summary | Adjusted P Value |  |  |  |
| --- | --- | --- | --- | --- | --- | --- | --- | --- |
|  |  |  |  |  |  |  |  |  |
| Non pCR |  |  |  |  |  |  |  |  |
| Baseline vs. Surgery | 1,076 | -5,913 to 8,065 | No | ns | 0,8527 |  |  |  |
| Baseline vs. Post-surgery | 0,2580 | -1,707 to 2,223 | No | ns | 0,8894 |  |  |  |
| Surgery vs. Post-surgery | -0,8180 | -6,013 to 4,377 | No | ns | 0,8468 |  |  |  |
|  |  |  |  |  |  |  |  |  |
| pCR |  |  |  |  |  |  |  |  |
| Baseline vs. Surgery | -4,435 | -16,61 to 7,738 | No | ns | 0,5101 |  |  |  |
| Baseline vs. Post-surgery | -0,9600 | -2,940 to 1,020 | No | ns | 0,3363 |  |  |  |
| Surgery vs. Post-surgery | 3,475 | -7,008 to 13,96 | No | ns | 0,5657 |  |  |  |
|  |  |  |  |  |  |  |  |  |
|  |  |  |  |  |  |  |  |  |
| Test details | Mean 1 | Mean 2 | Mean Diff, | SE of diff, | N1 | N2 | q | DF |
|  |  |  |  |  |  |  |  |  |
| Non pCR |  |  |  |  |  |  |  |  |
| Baseline vs. Surgery | 5,404 | 4,328 | 1,076 | 1,961 | 5 | 5 | 0,7760 | 4,000 |
| Baseline vs. Post-surgery | 5,404 | 5,146 | 0,2580 | 0,5512 | 5 | 5 | 0,6619 | 4,000 |
| Surgery vs. Post-surgery | 4,328 | 5,146 | -0,8180 | 1,458 | 5 | 5 | 0,7936 | 4,000 |
|  |  |  |  |  |  |  |  |  |
| pCR |  |  |  |  |  |  |  |  |
| Baseline vs. Surgery | 2,903 | 7,338 | -4,435 | 3,741 | 6 | 6 | 1,677 | 5,000 |
| Baseline vs. Post-surgery | 2,903 | 3,863 | -0,9600 | 0,6085 | 6 | 6 | 2,231 | 5,000 |
| Surgery vs. Post-surgery | 7,338 | 3,863 | 3,475 | 3,222 | 6 | 6 | 1,525 | 5,000 |

##### pCR vs non-pCR comparison

| Šídák's multiple comparisons test | Mean Diff, | 95,00% CI of diff, | Below threshold? | Summary | Adjusted P Value |  |  |  |
| --- | --- | --- | --- | --- | --- | --- | --- | --- |
|  |  |  |  |  |  |  |  |  |
| Non pCR - pCR |  |  |  |  |  |  |  |  |
| Baseline | 2,501 | -6,781 to 11,78 | No | ns | 0,7444 |  |  |  |
| Surgery | -3,010 | -17,06 to 11,03 | No | ns | 0,8841 |  |  |  |
| Post-surgery | 1,283 | -6,669 to 9,234 | No | ns | 0,9387 |  |  |  |
|  |  |  |  |  |  |  |  |  |
|  |  |  |  |  |  |  |  |  |
| Test details | Mean 1 | Mean 2 | Mean Diff, | SE of diff, | N1 | N2 | t | DF |
|  |  |  |  |  |  |  |  |  |
| Non pCR - pCR |  |  |  |  |  |  |  |  |
| Baseline | 5,404 | 2,903 | 2,501 | 2,478 | 5 | 6 | 1,009 | 4,387 |
| Surgery | 4,328 | 7,338 | -3,010 | 4,333 | 5 | 6 | 0,6948 | 6,173 |
| Post-surgery | 5,146 | 3,863 | 1,283 | 2,342 | 5 | 6 | 0,5477 | 5,444 |

### Treg at 3 timepoints, test differences between immune checkpoints (Fig 4B)

#### Treg - LAG3

##### Timepoint comparison

| Tukey's multiple comparisons test | Mean Diff, | 95,00% CI of diff, | Below threshold? | Summary | Adjusted P Value |  |  |  |
| --- | --- | --- | --- | --- | --- | --- | --- | --- |
|  |  |  |  |  |  |  |  |  |
| Baseline vs. Surgery | -0,4355 | -1,787 to 0,9160 | No | ns | 0,6624 |  |  |  |
| Baseline vs. Post-surgery | -1,105 | -3,103 to 0,8923 | No | ns | 0,3243 |  |  |  |
| Surgery vs. Post-surgery | -0,6700 | -3,682 to 2,342 | No | ns | 0,8181 |  |  |  |
|  |  |  |  |  |  |  |  |  |
|  |  |  |  |  |  |  |  |  |
| Test details | Mean 1 | Mean 2 | Mean Diff, | SE of diff, | N1 | N2 | q | DF |
|  |  |  |  |  |  |  |  |  |
| Baseline vs. Surgery | 1,812 | 2,247 | -0,4355 | 0,4930 | 11 | 11 | 1,249 | 10,00 |
| Baseline vs. Post-surgery | 1,812 | 2,917 | -1,105 | 0,7288 | 11 | 11 | 2,145 | 10,00 |
| Surgery vs. Post-surgery | 2,247 | 2,917 | -0,6700 | 1,099 | 11 | 11 | 0,8624 | 10,00 |

| Tukey's multiple comparisons test | Mean Diff, | 95,00% CI of diff, | Below threshold? | Summary | Adjusted P Value |  |  |  |
| --- | --- | --- | --- | --- | --- | --- | --- | --- |
|  |  |  |  |  |  |  |  |  |
| Non pCR |  |  |  |  |  |  |  |  |
| Baseline vs. Surgery | 0,3080 | -2,919 to 3,535 | No | ns | 0,9391 |  |  |  |
| Baseline vs. Post-surgery | -2,186 | -6,839 to 2,467 | No | ns | 0,3196 |  |  |  |
| Surgery vs. Post-surgery | -2,494 | -9,660 to 4,672 | No | ns | 0,4940 |  |  |  |
|  |  |  |  |  |  |  |  |  |
| pCR |  |  |  |  |  |  |  |  |
| Baseline vs. Surgery | -1,055 | -2,419 to 0,3090 | No | ns | 0,1137 |  |  |  |
| Baseline vs. Post-surgery | -0,2050 | -2,398 to 1,988 | No | ns | 0,9508 |  |  |  |
| Surgery vs. Post-surgery | 0,8500 | -1,901 to 3,601 | No | ns | 0,6052 |  |  |  |
|  |  |  |  |  |  |  |  |  |
|  |  |  |  |  |  |  |  |  |
| Test details | Mean 1 | Mean 2 | Mean Diff, | SE of diff, | N1 | N2 | q | DF |
|  |  |  |  |  |  |  |  |  |
| Non pCR |  |  |  |  |  |  |  |  |
| Baseline vs. Surgery | 2,030 | 1,722 | 0,3080 | 0,9054 | 5 | 5 | 0,4811 | 4,000 |
| Baseline vs. Post-surgery | 2,030 | 4,216 | -2,186 | 1,306 | 5 | 5 | 2,368 | 4,000 |
| Surgery vs. Post-surgery | 1,722 | 4,216 | -2,494 | 2,011 | 5 | 5 | 1,754 | 4,000 |
|  |  |  |  |  |  |  |  |  |
| pCR |  |  |  |  |  |  |  |  |
| Baseline vs. Surgery | 1,630 | 2,685 | -1,055 | 0,4192 | 6 | 6 | 3,559 | 5,000 |
| Baseline vs. Post-surgery | 1,630 | 1,835 | -0,2050 | 0,6738 | 6 | 6 | 0,4302 | 5,000 |
| Surgery vs. Post-surgery | 2,685 | 1,835 | 0,8500 | 0,8453 | 6 | 6 | 1,422 | 5,000 |

##### pCR vs non-pCR comparison

| Šídák's multiple comparisons test | Mean Diff, | 95,00% CI of diff, | Below threshold? | Summary | Adjusted P Value |  |  |  |
| --- | --- | --- | --- | --- | --- | --- | --- | --- |
|  |  |  |  |  |  |  |  |  |
| Non pCR - pCR |  |  |  |  |  |  |  |  |
| Baseline | 0,4000 | -1,120 to 1,920 | No | ns | 0,8208 |  |  |  |
| Surgery | -0,9630 | -3,552 to 1,626 | No | ns | 0,6567 |  |  |  |
| Post-surgery | 2,381 | -4,039 to 8,801 | No | ns | 0,5587 |  |  |  |
|  |  |  |  |  |  |  |  |  |
|  |  |  |  |  |  |  |  |  |
| Test details | Mean 1 | Mean 2 | Mean Diff, | SE of diff, | N1 | N2 | t | DF |
|  |  |  |  |  |  |  |  |  |
| Non pCR - pCR |  |  |  |  |  |  |  |  |
| Baseline | 2,030 | 1,630 | 0,4000 | 0,4835 | 5 | 6 | 0,8273 | 6,790 |
| Surgery | 1,722 | 2,685 | -0,9630 | 0,8712 | 5 | 6 | 1,105 | 8,361 |
| Post-surgery | 4,216 | 1,835 | 2,381 | 1,761 | 5 | 6 | 1,352 | 4,630 |

#### Treg - TIGIT

##### Timepoint comparison

| Tukey's multiple comparisons test | Mean Diff, | 95,00% CI of diff, | Below threshold? | Summary | Adjusted P Value |  |  |  |
| --- | --- | --- | --- | --- | --- | --- | --- | --- |
|  |  |  |  |  |  |  |  |  |
| Baseline vs. Surgery | 2,982 | -1,149 to 7,113 | No | ns | 0,1679 |  |  |  |
| Baseline vs. Post-surgery | -5,482 | -13,61 to 2,648 | No | ns | 0,2038 |  |  |  |
| Surgery vs. Post-surgery | -8,464 | -17,36 to 0,4330 | No | ns | 0,0622 |  |  |  |
|  |  |  |  |  |  |  |  |  |
|  |  |  |  |  |  |  |  |  |
| Test details | Mean 1 | Mean 2 | Mean Diff, | SE of diff, | N1 | N2 | q | DF |
|  |  |  |  |  |  |  |  |  |
| Baseline vs. Surgery | 60,65 | 57,66 | 2,982 | 1,507 | 11 | 11 | 2,798 | 10,00 |
| Baseline vs. Post-surgery | 60,65 | 66,13 | -5,482 | 2,966 | 11 | 11 | 2,614 | 10,00 |
| Surgery vs. Post-surgery | 57,66 | 66,13 | -8,464 | 3,245 | 11 | 11 | 3,688 | 10,00 |

| Tukey's multiple comparisons test | Mean Diff, | 95,00% CI of diff, | Below threshold? | Summary | Adjusted P Value |  |  |  |
| --- | --- | --- | --- | --- | --- | --- | --- | --- |
|  |  |  |  |  |  |  |  |  |
| Non pCR |  |  |  |  |  |  |  |  |
| Baseline vs. Surgery | 3,540 | -4,845 to 11,92 | No | ns | 0,3803 |  |  |  |
| Baseline vs. Post-surgery | -7,000 | -26,41 to 12,41 | No | ns | 0,4731 |  |  |  |
| Surgery vs. Post-surgery | -10,54 | -35,73 to 14,65 | No | ns | 0,3857 |  |  |  |
|  |  |  |  |  |  |  |  |  |
| pCR |  |  |  |  |  |  |  |  |
| Baseline vs. Surgery | 2,517 | -4,417 to 9,451 | No | ns | 0,5124 |  |  |  |
| Baseline vs. Post-surgery | -4,217 | -15,38 to 6,947 | No | ns | 0,4883 |  |  |  |
| Surgery vs. Post-surgery | -6,733 | -13,23 to -0,2344 | Yes | * | 0,0441 |  |  |  |
|  |  |  |  |  |  |  |  |  |
|  |  |  |  |  |  |  |  |  |
| Test details | Mean 1 | Mean 2 | Mean Diff, | SE of diff, | N1 | N2 | q | DF |
|  |  |  |  |  |  |  |  |  |
| Non pCR |  |  |  |  |  |  |  |  |
| Baseline vs. Surgery | 65,82 | 62,28 | 3,540 | 2,353 | 5 | 5 | 2,128 | 4,000 |
| Baseline vs. Post-surgery | 65,82 | 72,82 | -7,000 | 5,446 | 5 | 5 | 1,818 | 4,000 |
| Surgery vs. Post-surgery | 62,28 | 72,82 | -10,54 | 7,069 | 5 | 5 | 2,109 | 4,000 |
|  |  |  |  |  |  |  |  |  |
| pCR |  |  |  |  |  |  |  |  |
| Baseline vs. Surgery | 56,33 | 53,82 | 2,517 | 2,131 | 6 | 6 | 1,670 | 5,000 |
| Baseline vs. Post-surgery | 56,33 | 60,55 | -4,217 | 3,431 | 6 | 6 | 1,738 | 5,000 |
| Surgery vs. Post-surgery | 53,82 | 60,55 | -6,733 | 1,997 | 6 | 6 | 4,768 | 5,000 |

##### pCR vs non-pCR comparison

| Šídák's multiple comparisons test | Mean Diff, | 95,00% CI of diff, | Below threshold? | Summary | Adjusted P Value |  |  |  |
| --- | --- | --- | --- | --- | --- | --- | --- | --- |
|  |  |  |  |  |  |  |  |  |
| Non pCR - pCR |  |  |  |  |  |  |  |  |
| Baseline | 9,487 | -2,534 to 21,51 | No | ns | 0,1314 |  |  |  |
| Surgery | 8,463 | -4,917 to 21,84 | No | ns | 0,2593 |  |  |  |
| Post-surgery | 12,27 | -11,92 to 36,46 | No | ns | 0,4001 |  |  |  |
|  |  |  |  |  |  |  |  |  |
|  |  |  |  |  |  |  |  |  |
| Test details | Mean 1 | Mean 2 | Mean Diff, | SE of diff, | N1 | N2 | t | DF |
|  |  |  |  |  |  |  |  |  |
| Non pCR - pCR |  |  |  |  |  |  |  |  |
| Baseline | 65,82 | 56,33 | 9,487 | 4,061 | 5 | 6 | 2,336 | 8,515 |
| Surgery | 62,28 | 53,82 | 8,463 | 4,499 | 5 | 6 | 1,881 | 8,344 |
| Post-surgery | 72,82 | 60,55 | 12,27 | 7,712 | 5 | 6 | 1,591 | 6,840 |

#### Treg - CTLA4

##### Timepoint comparison

| Tukey's multiple comparisons test | Mean Diff, | 95,00% CI of diff, | Below threshold? | Summary | Adjusted P Value |  |  |  |
| --- | --- | --- | --- | --- | --- | --- | --- | --- |
|  |  |  |  |  |  |  |  |  |
| Baseline vs. Surgery | -0,2455 | -11,07 to 10,58 | No | ns | 0,9979 |  |  |  |
| Baseline vs. Post-surgery | -6,173 | -14,37 to 2,023 | No | ns | 0,1473 |  |  |  |
| Surgery vs. Post-surgery | -5,927 | -16,12 to 4,268 | No | ns | 0,2925 |  |  |  |
|  |  |  |  |  |  |  |  |  |
|  |  |  |  |  |  |  |  |  |
| Test details | Mean 1 | Mean 2 | Mean Diff, | SE of diff, | N1 | N2 | q | DF |
|  |  |  |  |  |  |  |  |  |
| Baseline vs. Surgery | 81,39 | 81,64 | -0,2455 | 3,949 | 11 | 11 | 0,08790 | 10,00 |
| Baseline vs. Post-surgery | 81,39 | 87,56 | -6,173 | 2,990 | 11 | 11 | 2,920 | 10,00 |
| Surgery vs. Post-surgery | 81,64 | 87,56 | -5,927 | 3,719 | 11 | 11 | 2,254 | 10,00 |

| Tukey's multiple comparisons test | Mean Diff, | 95,00% CI of diff, | Below threshold? | Summary | Adjusted P Value |  |  |  |
| --- | --- | --- | --- | --- | --- | --- | --- | --- |
|  |  |  |  |  |  |  |  |  |
| Non pCR |  |  |  |  |  |  |  |  |
| Baseline vs. Surgery | 3,440 | -22,18 to 29,06 | No | ns | 0,8848 |  |  |  |
| Baseline vs. Post-surgery | -3,340 | -8,775 to 2,095 | No | ns | 0,1867 |  |  |  |
| Surgery vs. Post-surgery | -6,780 | -36,82 to 23,26 | No | ns | 0,7206 |  |  |  |
|  |  |  |  |  |  |  |  |  |
| pCR |  |  |  |  |  |  |  |  |
| Baseline vs. Surgery | -3,317 | -17,34 to 10,71 | No | ns | 0,7362 |  |  |  |
| Baseline vs. Post-surgery | -8,533 | -25,99 to 8,920 | No | ns | 0,3313 |  |  |  |
| Surgery vs. Post-surgery | -5,217 | -10,97 to 0,5354 | No | ns | 0,0695 |  |  |  |
|  |  |  |  |  |  |  |  |  |
|  |  |  |  |  |  |  |  |  |
| Test details | Mean 1 | Mean 2 | Mean Diff, | SE of diff, | N1 | N2 | q | DF |
|  |  |  |  |  |  |  |  |  |
| Non pCR |  |  |  |  |  |  |  |  |
| Baseline vs. Surgery | 90,06 | 86,62 | 3,440 | 7,188 | 5 | 5 | 0,6768 | 4,000 |
| Baseline vs. Post-surgery | 90,06 | 93,40 | -3,340 | 1,525 | 5 | 5 | 3,097 | 4,000 |
| Surgery vs. Post-surgery | 86,62 | 93,40 | -6,780 | 8,429 | 5 | 5 | 1,138 | 4,000 |
|  |  |  |  |  |  |  |  |  |
| pCR |  |  |  |  |  |  |  |  |
| Baseline vs. Surgery | 74,17 | 77,48 | -3,317 | 4,311 | 6 | 6 | 1,088 | 5,000 |
| Baseline vs. Post-surgery | 74,17 | 82,70 | -8,533 | 5,364 | 6 | 6 | 2,250 | 5,000 |
| Surgery vs. Post-surgery | 77,48 | 82,70 | -5,217 | 1,768 | 6 | 6 | 4,173 | 5,000 |

##### pCR vs non-pCR comparison

| Šídák's multiple comparisons test | Mean Diff, | 95,00% CI of diff, | Below threshold? | Summary | Adjusted P Value |  |  |  |
| --- | --- | --- | --- | --- | --- | --- | --- | --- |
|  |  |  |  |  |  |  |  |  |
| Non pCR - pCR |  |  |  |  |  |  |  |  |
| Baseline | 15,89 | -4,135 to 35,92 | No | ns | 0,1202 |  |  |  |
| Surgery | 9,137 | -19,96 to 38,23 | No | ns | 0,6924 |  |  |  |
| Post-surgery | 10,70 | -2,027 to 23,43 | No | ns | 0,1041 |  |  |  |
|  |  |  |  |  |  |  |  |  |
|  |  |  |  |  |  |  |  |  |
| Test details | Mean 1 | Mean 2 | Mean Diff, | SE of diff, | N1 | N2 | t | DF |
|  |  |  |  |  |  |  |  |  |
| Non pCR - pCR |  |  |  |  |  |  |  |  |
| Baseline | 90,06 | 74,17 | 15,89 | 6,285 | 5 | 6 | 2,529 | 6,502 |
| Surgery | 86,62 | 77,48 | 9,137 | 8,406 | 5 | 6 | 1,087 | 5,193 |
| Post-surgery | 93,40 | 82,70 | 10,70 | 4,310 | 5 | 6 | 2,483 | 8,598 |

| Unpaired t test |  |
| --- | --- |
| P value | 0,0197 |
| P value summary | * |
| Significantly different (P < 0.05)? | Yes |
| One- or two-tailed P value? | Two-tailed |
| t, df | t=3,766, df=4 |
